# Process and costs for readiness to safely implement immediate kangaroo mother care: a mixed methods evaluation from the OMWaNA trial at five hospitals in Uganda

**DOI:** 10.1186/s12913-023-09624-z

**Published:** 2023-06-10

**Authors:** Melissa M. Medvedev, Victor Tumukunde, Charity Kirabo-Nagemi, Giulia Greco, Ivan Mambule, Kenneth Katumba, Peter Waiswa, Cally J. Tann, Diana Elbourne, Elizabeth Allen, Elizabeth Ekirapa-Kiracho, Catherine Pitt, Joy E. Lawn

**Affiliations:** 1 Department of Pediatrics, University of California San Francisco, 550 16th St., Box 1224, San Francisco, CA 94158 USA; 2 Maternal, Adolescent, Reproductive & Child Health Centre, London School of Hygiene & Tropical Medicine, Keppel St., London, WC1E 7HT UK; 3 Medical Research Council/Uganda Virus Research Institute and London School of Hygiene & Tropical Medicine Uganda Research Unit, P.O. Box 49, Entebbe, Uganda; 4 Department of Global Health and Development, London School of Hygiene & Tropical Medicine, 15-17 Tavistock Pl., London, WC1H 9SH UK; 5 Department of Health Policy, Planning and Management, School of Public Health, Makerere University, New Mulago Hill Rd., Kampala, Uganda; 6 Department of Public Health Sciences, Karolinska Institutet, 171 77 Stockholm, Sweden; 7 Department of Neonatal Medicine, University College London Hospital, 235 Euston Rd, London, NW1 2BU UK; 8 Department of Medical Statistics, London School of Hygiene & Tropical Medicine, Keppel St., London, WC1E 7HT UK

**Keywords:** Kangaroo mother care, Preterm, Low birthweight, Newborn care, Implementation, Intervention costs, Service readiness

## Abstract

**Background:**

Preterm birth complications result in > 1 million child deaths annually, mostly in low- and middle-income countries. A World Health Organisation (WHO)-led trial in hospitals with intensive care reported reduced mortality within 28 days among newborns weighing 1000–1799 g who received immediate kangaroo mother care (iKMC) compared to those who received standard care. Evidence is needed regarding the process and costs of implementing iKMC, particularly in non-intensive care settings.

**Methods:**

We describe actions undertaken to implement iKMC, estimate financial and economic costs of essential resources and infrastructure improvements, and assess readiness for newborn care after these improvements at five Ugandan hospitals participating in the OMWaNA trial. We estimated costs from a health service provider perspective and explored cost drivers and cost variation across hospitals. We assessed readiness to deliver small and sick newborn care (WHO level-2) using a tool developed by Newborn Essential Solutions and Technologies and the United Nations Children’s Fund.

**Results:**

Following the addition of space to accommodate beds for iKMC, floor space in the neonatal units ranged from 58 m^2^ to 212 m^2^. Costs of improvements were lowest at the national referral hospital (financial: $31,354; economic: $45,051; 2020 USD) and varied across the four smaller hospitals (financial: $68,330-$95,796; economic: $99,430-$113,881). In a standardised 20-bed neonatal unit offering a level of care comparable to the four smaller hospitals, the total financial cost could be in the range of $70,000 to $80,000 if an existing space could be repurposed or remodelled, or $95,000 if a new unit needed to be constructed. Even after improvements, the facility assessments demonstrated broad variability in laboratory and pharmacy capacity as well as the availability of essential equipment and supplies.

**Conclusions:**

These five Ugandan hospitals required substantial resource inputs to allow safe implementation of iKMC. Before widespread scale-up of iKMC, the affordability and efficiency of this investment must be assessed, considering variation in costs across hospitals and levels of care. These findings should help inform planning and budgeting as well as decisions about if, where, and how to implement iKMC, particularly in settings where space, devices, and specialised staff for newborn care are unavailable.

**Trial registration:**

ClinicalTrials.gov, NCT02811432. Registered: 23 June 2016.

**Supplementary Information:**

The online version contains supplementary material available at 10.1186/s12913-023-09624-z.

## Background

Each year, nearly 15 million babies are born preterm (< 37 weeks’ gestation) [1]. Preterm birth rates are rising, with the highest risk in sub-Saharan Africa [1, 2]. Complications of prematurity result in > 1 million child deaths annually [3], mostly in low- and middle-income countries (LMIC). At least 1 million survivors of preterm birth suffer from moderate or severe neurodevelopmental impairment [4]. Furthermore, neonatal conditions are the leading cause of disability-adjusted life-years worldwide, contributing 7% in 2019 [5]. Addressing the global burden of preterm birth is crucial to achieving Sustainable Development Goal 3 to ensure healthy lives and promote wellbeing [6]. Given slow progress in neonatal mortality reduction, the United Nations launched a new Every Newborn Action Plan target to ensure that by 2025, 80% of districts have a hospital with a newborn special care unit, including thermal support with kangaroo mother care (KMC); assisted feeding and intravenous (IV) fluids; safe oxygen administration; and management of sepsis, jaundice, apnoea, and respiratory distress, including with continuous positive airway pressure (CPAP) [7]. Estimates suggest that achieving 95% coverage of high-quality special care (level-2) or intensive care (level-3) in 81 high-burden countries could prevent 750,000 neonatal deaths annually and dramatically reduce mortality due to prematurity [8].

KMC is an evidence-based intervention involving prolonged skin-to-skin contact, promotion of exclusive breastmilk feeding, facilitation of early hospital discharge, and adequate support and follow-up at home [9]. KMC is associated with decreased mortality, sepsis, hypothermia, and hypoglycaemia [10, 11], as well as possible long-term benefits to intellectual quotient [12], when initiated in stabilised babies. However, the majority of neonatal deaths occur within 48 h of birth [13], before clinical stabilisation. Establishing the mortality impact of KMC initiated before stabilisation is therefore a research priority [14, 15], which several recently completed randomised controlled trials (RCT) sought to address [16–18]. The World Health Organisation (WHO) Immediate KMC Study, conducted in five tertiary-level hospitals with intensive care, reported reduced mortality at 28 days among newborns weighing 1000–1799 g (g) who received immediate KMC (iKMC) relative to those who received standard care with KMC initiated after stabilisation [17]. These promising findings have spurred calls for widespread adoption of iKMC, even though more than three-quarters of neonates in sub-Saharan Africa and southern Asia lack access to intensive care [19]. In November 2022, the WHO released new guidelines for the care of preterm and low birthweight (< 2500 g) infants, which include a shift to recommending that iKMC be initiated within 24 h of birth, before stabilisation, at all levels of facility-based newborn care [20]. These guidelines also highlight the need for special and intensive care units that care for babies and mothers together. Evidence is therefore needed regarding the process and costs of successfully implementing iKMC and how these vary across contexts, including in hospitals without neonatal intensive care [16, 18].

We aim to inform decisions about if, where, and how to implement iKMC by analysing baseline data from OMWaNA, a pragmatic RCT evaluating the mortality impact of KMC initiated before stabilisation compared to standard care in Uganda. Specifically, the objectives of this analysis are to: i) describe the actions undertaken to safely implement facility-based iKMC before the start of the trial; ii) estimate the financial and economic costs of these essential resources and infrastructure improvements; and iii) assess service readiness for small and sick newborn care following these improvements at five hospitals in Uganda. Our purpose is to provide evidence on the process and costs of the required improvements to allow safe implementation of iKMC. We consider the generalisability of our findings to other hospitals in LMICs and the implications for scale-up of iKMC in contexts without neonatal intensive care.

## Methods

The protocol for the OMWaNA trial has previously been published [16]. This analysis has been reported in accordance with the Standards for Reporting Implementation Studies (StaRI) statement [21]. The StaRI checklist is available in Additional file 1.

### Study setting

The OMWaNA trial was led by the Medical Research Council/Uganda Virus Research Institute (MRC/UVRI) and London School of Hygiene & Tropical Medicine (LSHTM) Uganda Research Unit in Entebbe, in collaboration with Makerere University and LSHTM. The trial was conducted in five government hospitals in Uganda:

• Entebbe Regional Referral Hospital (Hospital-1)
• Iganga District Hospital (Hospital-2)
• Jinja Regional Referral Hospital (Hospital-3)
• Kawempe National Referral Hospital (Hospital-4)

• Masaka Regional Referral Hospital (Hospital-5)

**Fig. 1:**
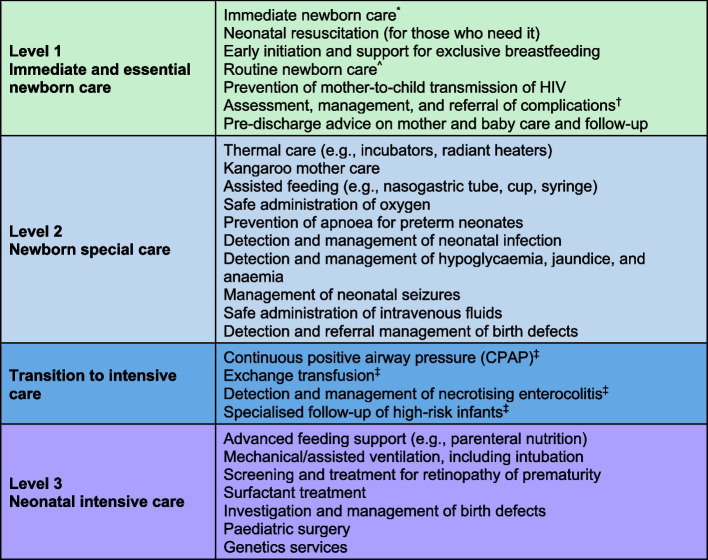
Inpatient care for small and sick newborns: WHO standards by level of care. HIV = human immunodeficiency virus. ^*^Including drying, skin-to-skin contact with the mother, delayed cord clamping, and hygienic cord care. ^^^Including Vitamin K, eye care, vaccinations, weighing, and clinical examinations. ^†^Including bacterial infections, jaundice, diarrhoea, feeding problems, birth defects, and other problems. ^‡^Hospitals providing special care should introduce these interventions before upgrading to intensive care. Figure adapted from ‘Survive and Thrive: Transforming care for every small and sick newborn’ (2019) [8]

Prior to the OMWaNA study, each hospital had a newborn special care unit, hereafter referred to as ‘neonatal unit,’ that accepted referrals from their respective catchment area. The availability of equipment in these facilities varied, but all had incubators and/or radiant heaters, oxygen supply, and standard operating procedures for clinical management, including respiratory distress, apnoea, infection, seizures, hypothermia, and hypoglycaemia. Most facilities met WHO level-2 criteria (Fig. 1), although at the start of the study, few were consistently practicing CPAP. Recruitment began at Hospitals 1, 2, 3, and 5 in November 2019. In March 2020, the Ugandan government designated Hospital-1 as a COVID-19 quarantine facility and recruitment was stopped at that site. Hospital-4 was subsequently added as a site, commencing recruitment in October 2020.

### Renovations and improvements to the neonatal units

We describe key infrastructure improvements and clinical equipment provided to health facilities prior to initiation of the trial based on study and hospital records. Overall infrastructure improvements are classified in the following categories:

• *Repurposing*: reallocation of existing space within the hospital to the neonatal unit or KMC area
• *Extension/remodelling*: major improvements to existing neonatal unit or KMC area, including construction of an extension to create additional space

• *Construction of new unit*: construction of a new neonatal unit, entirely separate from an existing unit

Estimates of neonatal unit floor space (metres squared, m^2^) after improvements are presented in total, per neonatal bed, and per neonatal admission. The latter were estimated using the total number of neonatal beds (cots, incubators, radiant heaters) and the number of annual neonatal admissions divided by 365 days, respectively.

### Financial and economic costs of infrastructure improvements and clinical equipment

We estimated the financial and wider economic costs of the essential resources provided to the five neonatal units to allow them to implement iKMC safely. Financial costs reflect actual monies paid (or expenditure). Economic costs reflect the full value of all resources used; they therefore encompass both financial costs and the value of donated resources and volunteer time. Costs were estimated from a health service provider perspective. Data on resource use and unit and total costs were collected from hospital records and the project and accounting records of the OMWaNA trial at MRC/UVRI, then collated into an Excel-based costing tool for each hospital. We costed inputs and resources used for: 1) planning and design, 2) infrastructure improvements, 3) clinical equipment, and 4) training hospital staff on KMC and clinical guidelines (Table 1).

**Table 1:** Cost categories

**• Planning and design costs**: staff time during contract drafting, inception, initial and interim site and closure meetings, tendering, scoping, site survey, and inspection; transportation costs for site survey, inspection, and meetings
**• Infrastructure improvement costs**: financial costs of materials, labour, and transportation used in the construction, remodelling, and repurposing of space for neonatal units; rental of office space at MRC/UVRI for 3 months of setup activities
**• Equipment costs**: purchase of clinical equipment; clinical supplies were excluded from this analysis
**• Training costs**: trainers’ time spent preparing for and attending the training; monies spent on training materials, lunch, and transport refund for trainees; accommodation, meals, and transport for trainers

To estimate the economic costs of time spent by hospital and MRC staff during planning, design, and training activities, key informants were interviewed at MRC/UVRI and at the hospitals. We assumed that the opportunity cost of staff members’ time was equivalent to their pro-rated salaries in terms of hours spent conducting the planning, design, and training activities. The duration of time spent by hospital and MRC staff was based on the MRC engineer’s records of meetings and other activities. Salaries were obtained from project accounts for MRC staff and the Uganda Health Service Commission Circular No. 8 salary scale (2020) for hospital staff. We assumed an 8-h workday and 22 working days per month. The financial costs charged to the project for planning and design activities were estimated using a rate of United States Dollars (USD) $21 per person-hour. The financial costs of using MRC/project vehicles were estimated at a rate of $1.22 per kilometre travelled to and from the hospital sites. The rate covered fuel and maintenance while the opportunity cost was assumed to be equal to the cost of hire, fuel, and maintenance for comparable vehicles. The opportunity cost of renting training rooms was estimated at a daily rate of $41 per room, using the Uganda Public Procurement and Disposal of Public Assets price list (2020).

Floor space at the five hospitals was expanded in different ways. Where a new neonatal unit or extension to an existing unit was constructed, the financial costs of these building works were considered also to reflect the economic costs of this investment. Where existing space within the hospital was reallocated to the neonatal unit, this renovated space did not incur a financial cost; we therefore estimated the total economic costs based on the financial cost per m^2^ to construct a new space of equivalent size. Additional economic costs for donated space reflect the difference between this estimate of the total economic costs and the actual financial costs incurred for renovation activities.

We present the total financial costs and annualised economic costs of improvements per hospital, per neonatal bed, and per annual neonatal admission. Understanding total financial costs is important for planning and understanding budget impact. Annualised economic costs take into account the depreciation of capital inputs as well as the value of alternative (foregone) opportunities for using the resources tied up in the capital inputs (i.e., opportunity cost) [22]. Annualised economic costs are important for informing understanding of the efficiency of the investment compared with other potential uses of those resources, considering the expected lifespans of the different elements of the up-front investments. We explore key cost drivers and variation in costs across the hospitals.

Costs are presented in 2020 USD and Ugandan Shillings (UGX; see Supplementary Table 1, Additional file 2). No inflation adjustments were necessary, as all resources were purchased or used in the same year. Currencies were converted using World Bank average exchange rates for 2020 ($1 = UGX 3,641 = £0.72) [23]. Costs were annualised using a discount rate of 3% [24], and assumptions about the lifespan of capital improvements, equipment, and activities. The life expectancy of equipment was informed by interviewing officials from the National Medical Stores and Joint Medical Stores, which are the bodies mandated to procure medicines, supplies, and equipment in Uganda. Cost analyses were conducted in Microsoft Excel.

### Health facility assessments

Following completion of renovations and improvements, we assessed the readiness of the five hospitals to deliver care for small and sick newborns. We used a health facility assessment (HFA) tool that was developed by Newborn Essential Solutions and Technologies (NEST360), in partnership with the United Nations Children’s Fund (UNICEF), through a multi-stage process [25]. Briefly, a matrix of service readiness requirements was expanded to include 870 items [26], in line with WHO standards for improving the quality of neonatal care [27], then compared against existing obstetric and neonatal service assessment tools. A novel HFA tool was co-designed with four African government teams to collect data necessary for WHO level-2 care and enable data collection in one day. The resultant tool comprises four modules (facility and neonatal unit infrastructure; medical devices and supplies; human resources; information systems), with a total of 3,610 variables, restructured by WHO health system building block [19, 28]. Complete details regarding the development of this tool will be reported elsewhere [25].

Facility assessments took place at Hospitals 1, 2, 3, and 5 in February 2020 and at Hospital-4 in October 2020. All HFAs were conducted by the same team, comprised of one study medical officer, four study nurses, and one biomedical engineer from Uganda, who completed a 5-day training in January 2020. This training included a detailed review of objectives, tools, and data collection procedures, as well as practice conducting HFAs and collecting data using an Android tablet-based REDCap (Research Electronic Data Capture, Nashville, TN, USA) application. The data collectors confirmed the presence and functionality of items located in clinical areas, and orally asked pharmacy staff to assess the availability of drugs. Data from tablets were synchronised over a secure connection with the web-based REDCap database [29], hosted at the MRC/UVRI data centre. Data from the infrastructure and medical devices and supplies modules were summarised using descriptive statistics, including frequency, proportion, mean, standard deviation (SD), median, and interquartile range (IQR). Annual volumes of deliveries, admissions, referrals, and transfers reflect total numbers in the preceding calendar year. The results are organised by WHO health system building block. HFA analyses were conducted using Stata 15.1 (StataCorp, College Station, TX, USA).

## Results

### Actions undertaken to implement immediate KMC

Substantial infrastructure improvements and expansion of neonatal care capacity were required at all study hospitals to safely implement iKMC. The renovation process at the five study hospitals included the addition of floor space to improve the neonatal units and accommodate adult beds for KMC through repurposing or remodelling of existing space, or construction of a new unit. At Hospital-3, an extension to the existing neonatal unit was constructed, which increased floor space by 124% (Table 2). At Hospital-5, a new neonatal unit was constructed, which increased floor space by 98%. At Hospitals 1 and 2, existing space within the hospital was reallocated to the neonatal unit, increasing floor space by 18% and 142%, respectively. At Hospital-4, existing space within the neonatal unit was reallocated to the KMC area, but the floor space of the neonatal unit did not change.

**Table 2:** Floor space before and after renovation in neonatal units at five hospitals in Uganda

	**Hospital-1**	**Hospital-2**	**Hospital-3**	**Hospital-4**	**Hospital-5**
Type of hospital	Regional	District	Regional	National	Regional
Neonatal unit beds^a^, n	14	17	30	106	16
Average daily neonatal admissions^b^, n	1.4	2.6	6.7	18.6	1.0
Total floor space pre-renovation, m^2^	80	24	80	212	50
Floor space per bed pre-renovation^c^, m^2^	5.7	1.4	2.7	2.0	3.1
Floor space per daily neonatal admission pre-renovation^d^, m^2^	58.4	9.3	12.0	11.4	50.5
Total floor space post-renovation, m^2^	94	58	179	212	99
Floor space per bed post-renovation^c^, m^2^	6.7	3.4	6.0	2.0	6.2
Floor space per daily neonatal admission post-renovation^d^, m^2^	68.6	22.6	26.9	11.4	100.0
Change in total floor space, %	17.5	141.7	123.8	0	98.0
Change in floor space per bed^c^, %	17.5	142.9	122.2	0	100.0
Change in floor space per daily neonatal admission^d^, %	17.5	143.0	124.2	0	98.0
Financial cost of infrastructure improvements per m^2e^ (constant 2020 USD)	305.7	512.8	173.3	5.4	476.7

m^2^ = metres squared. USD = United States Dollars

^a^Total capacity of neonatal unit if one baby per bed (including cots, radiant heaters, and incubators)

^b^Calculated as the number of annual neonatal admissions divided by 365 days

^c^Calculated as the total floor space divided by the number of neonatal unit beds (including cots, incubators, and radiant heaters)

^d^Calculated as the total floor space divided by the daily average of annual neonatal admissions

^e^Calculated as the financial cost of infrastructure improvements divided by the total floor space post-renovation

Following these improvements, total floor space in the neonatal units ranged from 58 m^2^ at Hospital-2, a district-level facility, to 212 m^2^ at Hospital-4, a national referral facility (Table 2, see Additional file 3). Floor space per neonatal bed ranged from 2.0 m^2^ at Hospital-4 to 6.7 m^2^ at Hospital-1, a regional referral facility. Floor space per daily neonatal admission ranged from 11.4 m^2^ at Hospital-4 to 100.0 m^2^ at Hospital-5, a regional referral facility. Renovations also included the addition of offices for clinical staff (Hospitals 1, 2, 3, and 5), the addition of bathrooms and toilets for mothers and other caregivers (Hospitals 3 and 5), installation of sinks in clinical areas to promote infection prevention and control (Hospitals 1, 3, and 5), and installation of piped oxygen in the KMC area (Hospital-4).

Four members of staff from MRC/UVRI (one engineer, one trial coordinator, one site coordinator, and one procurement officer) were involved in planning, design, contract drafting, tendering, scoping, of the improvements, as well as initial and interim site and closure meetings, site survey, and inspection, supported by one driver from MRC/UVRI. An administrator from each of the five hospitals was involved in meetings, site survey, and inspection. The engineer, trial coordinator, site coordinator, procurement officer, hospital administrators, and drivers were estimated to have spent a total of 898, 290, 91, 456, 201, and 104 person-hours, respectively, on planning and design activities for all five sites.

All hospital staff completed a comprehensive, 5-day training programme on small and sick newborn care that was developed using established UNICEF and WHO protocols. This programme included Helping Babies Breathe, a neonatal resuscitation curriculum designed for low-resource settings [30, 31]. All hospitals were also provided with essential equipment and supplies to support the provision of KMC and small and sick newborn care (see Supplementary Table 2, Additional file 2).

### Financial and economic costs of infrastructure improvements and clinical equipment

#### Total costs of improvements

**Fig. 2:**
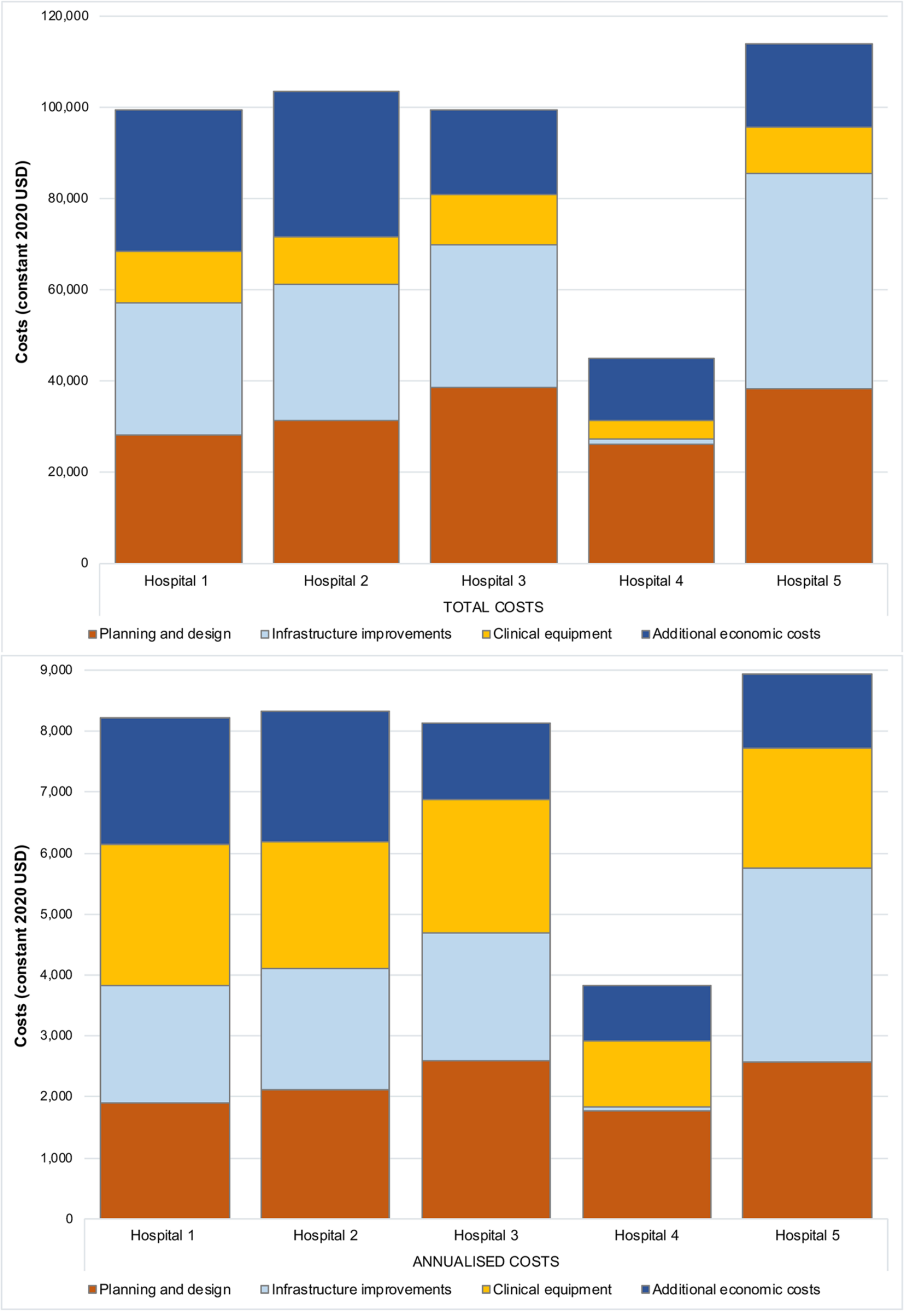
Key drivers of economic costs of improvements at the five Ugandan hospitals. The upper panel shows drivers of total economic costs, and the lower panel shows drivers of annualised economic costs

The overall economic cost of improvements in the five hospitals was $461,296, varying from $45,051 at Hospital-4 to $113,881 at Hospital-5 (Table 3). Additional economic costs (i.e., opportunity costs) comprised the largest share of total economic costs at Hospital-2 ($31,955, 31%) and Hospital-1 ($31,100, 31%), largely driven by the value of donated space (Fig. 2). Infrastructure improvements comprised the largest portion of total economic costs at Hospital-5 ($47,189, 41%). At Hospital-4, infrastructure improvement costs were minimal ($1,142, 3%) because the hospital had been recently constructed. Planning and design comprised the largest share of costs at Hospital-3 ($38,733, 39%) and Hospital-4 ($26,286, 58%). Costs of clinical equipment were lower at Hospital-4 ($3,926, 9%), which already had some of the necessary equipment, but similar across the other hospitals (range: $10,315, $11,307).

**Table 3:** Financial and economic costs of resources and infrastructure improvements to prepare for immediate KMC (all costs in USD, 2020)

	**Lifespan (years)**	**Hospital-1**		**Hospital-2**		**Hospital-3**		**Hospital-4**		**Hospital-5**		**ALL HOSPITALS**	
		**Total cost**	**Annualised cost**	**Total cost**	**Annualised cost**	**Total cost**	**Annualised cost**	**Total cost**	**Annualised cost**	**Total cost**	**Annualised cost**	**Total cost**	**Annualised cost**
***Financial costs***													
**Planning and design**													
Tendering and contracting	20	12,222	822	12,222	822	21,765	1,463	14,231	957	21,765	1,463	82,205	5,527
Scoping of work	20	5,016	337	5,016	337	1,005	68	1,005	68	1,005	68	13,047	878
Design	20	502	34	502	34	837	56	502	34	837	56	3,180	214
Site survey and inspection	20	1,507	101	1,507	101	1,507	101	1,507	101	1,507	101	7,535	505
Meetings	20	9,041	608	9,041	608	11,050	743	9,041	608	11,050	743	49,223	3,310
Transportation for site visits	20	NA	NA	3,156	212	2,569	173	NA	NA	2,128	143	7,853	528
**Sub-total (planning and design)**	**NA**	**28,288 (29)**	**1,902 (23)**	**31,444 (30)**	**2,114 (25)**	**38,733 (39)**	**2,604 (32)**	**26,286 (58)**	**1,768 (46)**	**38,292 (34)**	**2,574 (29)**	**163,043 (35)**	**10,962 (29)**
**Infrastructure improvements**													
Construction of new neonatal unit	20	NA	NA	NA	NA	NA	NA	NA	NA	47,045	3,162	47,045	3,162
Extension/remodelling of existing neonatal unit	20	NA	NA	NA	NA	30,881	2,076	1,142	77	NA	NA	32,023	2,153
Repurposing of space for neonatal unit	20	28,591	1,922	29,600	1,990	NA	NA	NA	NA	NA	NA	58,191	3,912
Rental of office space	20	144	10	144	10	144	10	NA	NA	144	10	576	40
**Sub-total (infrastructure improvements)**	**NA**	**28,735 (29)**	**1,932 (24)**	**29,744 (29)**	**2,000 (24)**	**31,025 (31)**	**2,086 (26)**	**1,142 (3)**	**77 (2)**	**47,189 (41)**	**3,172 (36)**	**137,835 (30)**	**9,267 (25)**
**Clinical equipment**^**a**^													
KMC adjustable bed^b^	5	476	104	1,189	260	1,189	260	2,326	508	1,189	260	6,369	1,392
Oxygen concentrator^c^	3	1,224	433	1,224	433	1,224	433	1,386	490	1,224	433	6,282	2,222
Masimo Rad-8^©^ pulse oximeter	7	7,225	1,160	4,516	725	5,419	870	0	0	4,516	725	21,676	3,480
Masimo LNC-04 patient cable	7	744	119	744	119	558	90	0	0	465	75	2,511	403
Glucose meter	3	30	11	30	11	30	11	0	0	30	11	120	44
Digital baby weighing scale^d^	3	857	303	857	303	854	302	0	0	857	303	3,425	1,211
Neonatal measuring mat^e^	3	47	17	47	17	47	17	0	0	47	17	188	68
Neonatal ventilation bag and mask	3	23	8	23	8	23	8	23	8	0	0	92	32
Digital axillary thermometer	3	9	3	9	3	9	3	0	0	9	3	36	12
Paediatric stethoscope	3	175	62	175	62	175	62	0	0	175	62	700	248
Training on KMC and clinical guidelines	35	306	14	1,336	62	1,374	64	0	0	1,803	84	4,819	224
Neonatal resuscitator	5	23	5	23	5	23	5	23	5	0	0	92	20
Penguin newborn suction	3	10	4	10	4	10	4	10	4	0	0	40	16
NeoNatalie^©^ manikin^f^	3	92	33	92	33	92	33	92	33	0	0	368	132
PreemieNatalie^©^ manikin^f^	3	66	23	66	23	66	23	66	23	0	0	264	92
**Sub-total (clinical equipment)**	**NA**	**11,307 (11)**	**2,299 (28)**	**10,341 (10)**	**2,068 (25)**	**11,093 (11)**	**2,185 (27)**	**3,926 (9)**	**1,071 (28)**	**10,315 (9)**	**1,973 (22)**	**46,982 (10)**	**9,596 (26)**
**Sub-total (financial cost)**	**NA**	**68,330 (69)**	**6,133 (75)**	**71,529 (69)**	**6,182 (74)**	**80,851 (81)**	**6,875 (85)**	**31,354 (70)**	**2,916 (76)**	**95,796 (84)**	**7,719 (86)**	**347,860 (75)**	**29,825 (80)**
***Additional economic costs***													
Planning and design^g^	20	8,232	553	11,388	765	11,522	774	7,048	474	11,081	745	49,271	3,311
Training^h^	20	6,649	447	7,175	482	7,077	476	6,649	447	7,004	471	34,554	2,323
Donated space^i^	20	16,219	1,090	13,392	900	0	0	0	0	0	0	29,611	1,990
**Sub-total (additional economic costs)**	**NA**	**31,100 (31)**	**2,090 (25)**	**31,955 (31)**	**2,147 (26)**	**18,599 (19)**	**1,250 (15)**	**13,697 (30)**	**921 (24)**	**18,085 (16)**	**1,216 (14)**	**113,436 (25)**	**7,624 (20)**
***Total economic cost***	**NA**	**99,430**	**8,223**	**103,484**	**8,329**	**99,450**	**8,125**	**45,051**	**3,837**	**113,881**	**8,935**	**461,296**	**37,449**

*KMC* Kangaroo mother care, *NA* Not applicable, *USD* United States Dollars. Costs were annualised using a discount rate of 3%, [24] and assumptions about the lifespan of capital improvements, equipment, and activities. Column percentages indicate the proportion of total economic costs for each hospital

^a^Clinical equipment and durable goods that were essential to allow safe implementation of immediate KMC, in accordance with the OMWaNA trial protocol [16]

^b^E3A India Narag adjustable bed, Crown Health Care Ltd., Kampala, Uganda

^c^USA Airsep oxygen concentrator, Crown Health Care Ltd., Kampala, Uganda

^d^Seca 384 digital weighing scale

^e^Seca 210 neonatal measuring mat

^f^Leardal NeoNatalie^©^ and PreemieNatalie^©^ neonatal resuscitation manikins

^g^Time spent by hospital staff away from normal duties to attend meetings or inspect the study sites

^h^Time spent by the trainers away from normal duties to conduct staff training

^i^Calculated as the difference between financial costs of renovations and economic costs to construct a new space of equivalent size, estimated using the unit financial cost of improvements at Hospital-5

Infrastructure setup costs primarily reflected the building, remodelling, and repurposing of the neonatal units (Table 3). Specific resource use varied across hospitals, but included demolition works and the installation of swing doors, aluminium partitions, window blinds, worktops, and electrical fixtures at one or more hospitals. Rental of office space at MRC/UVRI was required for two staff members who coordinated planning, design, and setup activities; these costs were attributed equally across the four hospitals involved at the start of the trial. Clinical setup costs included the purchase of equipment and durable goods that were essential to allow safe implementation of iKMC, in accordance with the trial protocol [16]. These resources included adjustable KMC beds, oxygen concentrators, pulse oximeters, weighing scales, and resuscitation manikins (Table 3; Supplementary Table 2, Additional file 2). Consumable supplies, such as adhesive pulse oximetry sensors, glucose test strips, nasal cannulas, and KMC wraps were purchased but not included in the tables, as they are recurrent costs. Training costs varied according to the number of staff trained, their level of experience, the cost of training materials and meals, and the distance travelled by trainees to the training site. The number of staff trained ranged from 6 to 12 per site. Accommodation ($28 per person per night) and meal costs ($10 per person per day) for the trainers were similar across Hospitals 2, 3, and 5, but were lower at Hospital-1, which was located near MRC/UVRI offices, and at Hospital-4, which did not receive training because the trained staff from Hospital-1 were transferred to Hospital-4 following site closure. Transportation costs were higher for Hospital-2, which is the furthest from Entebbe/Kampala.

At Hospitals 3 and 5, where a new neonatal unit or an extension to an existing unit was constructed, the financial costs of these building works were considered to reflect the total economic costs of these investments. At Hospitals 1 and 2, where existing space within the hospital was reallocated to the neonatal unit, the total economic costs were based on the financial cost to construct a new space of equivalent size, estimated using the financial cost at Hospital-5 ($477 per m^2^; Table 2). Additional economic costs for donated space, which reflect the difference between the total financial costs and the actual financial costs of renovation activities, were $13,392 at Hospital-2 (34 m^2^) and $16,219 at Hospital-1 (94 m^2^; Table 3).

#### Annualised economic costs of improvements, unit costs, and cost variation

The annualised economic costs of improvements ranged from $3,837 at Hospital-4 to $8,935 at Hospital-5 (Table 3). Necessary improvements at Hospital-4, the national referral hospital, cost approximately 54% to 57% less in total annualised economic costs than at the other hospitals because it was a higher-level hospital requiring fewer renovations and less clinical equipment to meet the minimum standard. Per annual neonatal admission, these annualised economic costs ranged from a low of $1 at Hospital-4 to $25 at Hospital-5 (Table 4). Annualised costs per neonatal bed ranged from $36 at Hospital-4 to $587 at Hospital-1. Per neonatal bed, costs at Hospital-4 were 87 to 94% lower than at the other hospitals because these already lower costs of improvement were spread over a larger number of neonatal beds (*n* = 106) compared to the other hospitals (range: 14–30).

**Table 4:** Hospital characteristics and incremental costs of improvements per admission and per bed

	**Hospital-1**	**Hospital-2**	**Hospital-3**	**Hospital-4**	**Hospital-5**	**Total**
Type of hospital	Regional	District	Regional	National	Regional	NA
Urban versus rural	Urban	Urban	Urban	Urban	Urban	NA
Population of catchment area, n	500,000	2,000,000	4,500,000	45,741,000^a^	Unknown	NA
Type of improvement to increase space available for iKMC	Repurposing existing space	Repurposing existing space	Remodelling existing space	Remodelling existing space	Construction of new space	NA
Annual deliveries^b^, n	2,000	7,500	6,937	21,606	6,000	44,043
Annual neonatal admissions^b^, n	500	938	2,432	6,782	360	11,012
Annual neonatal referrals^bc^, n	60	90	978	1,019	30	2,177
Annual neonatal transfers^bd^, n	10	20	10	Unknown	10	50
Total hospital beds, n	500	100	500	300	200	1,600
Labour ward beds, n	7	25	6	73	25	136
Postnatal ward beds, n	20	0	21	128	25	194
Neonatal unit beds^e^, n	14	17	30	106	16	183
Neonates currently in neonatal unit^f^, n	8	3	14	80	14	119
Neonatal unit capacity filled, %	57	18	47	75	88	NA
Annualised economic cost of improvements per annual neonatal admissions^g^ (2020 USD)	16	9	3	1	25	54
Annualised economic cost of improvements per neonatal bed^h^ (2020 USD)	587	490	271	36	558	1,942
Total financial cost of improvements per neonatal bed^i^ (2020 USD)	4,881	4,208	2,695	296	5,987	18,067

*iKMC* immediate kangaroo mother care, *NA* Not applicable, *USD* United States Dollars, *no inflation adjustments*

^a^Hospital-4 is a national referral facility; thus, its catchment area encompasses the whole country; total population 45,741,000 in 2020 (World Bank)

^b^Figure reflects total number in the calendar year preceding the baseline health facility assessment

^c^Referral to the hospital from another health facility

^d^Transfer from the hospital to another health facility

^e^Total capacity of neonatal unit if one baby per bed (including cots, radiant heaters, and incubators)

^f^Number of neonates admitted at the time of the health facility assessment

^g^Calculated as the total annualised economic cost per hospital divided by the number of annual neonatal admissions. ^h^Calculated as the total annualised economic cost per hospital divided by the number of neonatal beds

^i^Calculated as the total financial cost per hospital divided by the number of neonatal unit beds

**Fig. 3:**
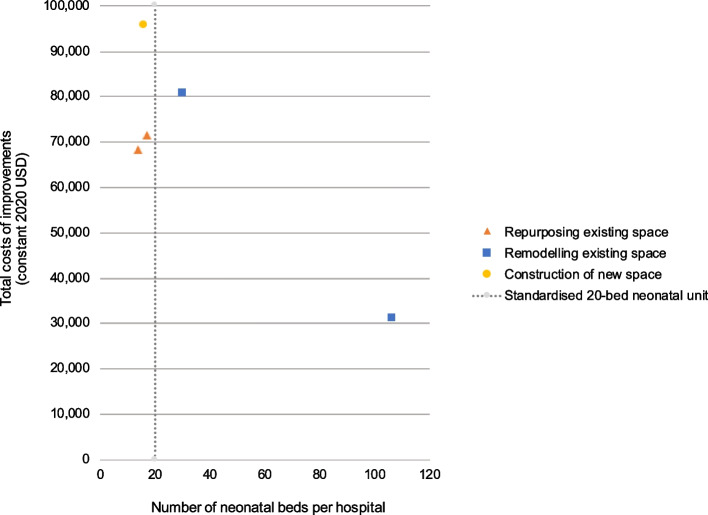
Total financial costs of improvements vs. the number of neonatal beds at the five Ugandan hospitals

For the four hospitals with broadly comparable numbers of neonatal beds, the costs of improvements varied (Fig. 3), with construction of a new unit in Hospital-5 (financial: $95,796; economic: $113,881) more expensive than remodelling at Hospital-3 (financial: $80,851; economic: $99,450), which in turn was more expensive than repurposing an existing space at Hospital-1 and Hospital-2 (financial: $68,330, $71,529; economic: $99,430, $103,484).

### Health facility assessments

Across all five hospitals, a median of 6,937 (IQR: 6,000–7,500; Table 4) babies were delivered annually. Delivery volumes were highest at Hospital-4 (*n* = 21,606) and lowest at Hospital-1 (*n* = 2,000). A median of 938 (IQR: 500–2,432) neonates were admitted annually across the five hospitals. The number of neonates admitted annually was highest at Hospital-4 (*n* = 6,782) and lowest at Hospital-5 (*n* = 360). A median of 90 (IQR: 60–978) neonates were referred to the five hospitals and 10 (IQR: 10–15) neonates were transferred from the five hospitals annually. The median number of total hospital beds and neonatal unit beds, respectively, were 300 (IQR: 200–500) and 17 (IQR: 16–30).

**Fig. 4:**
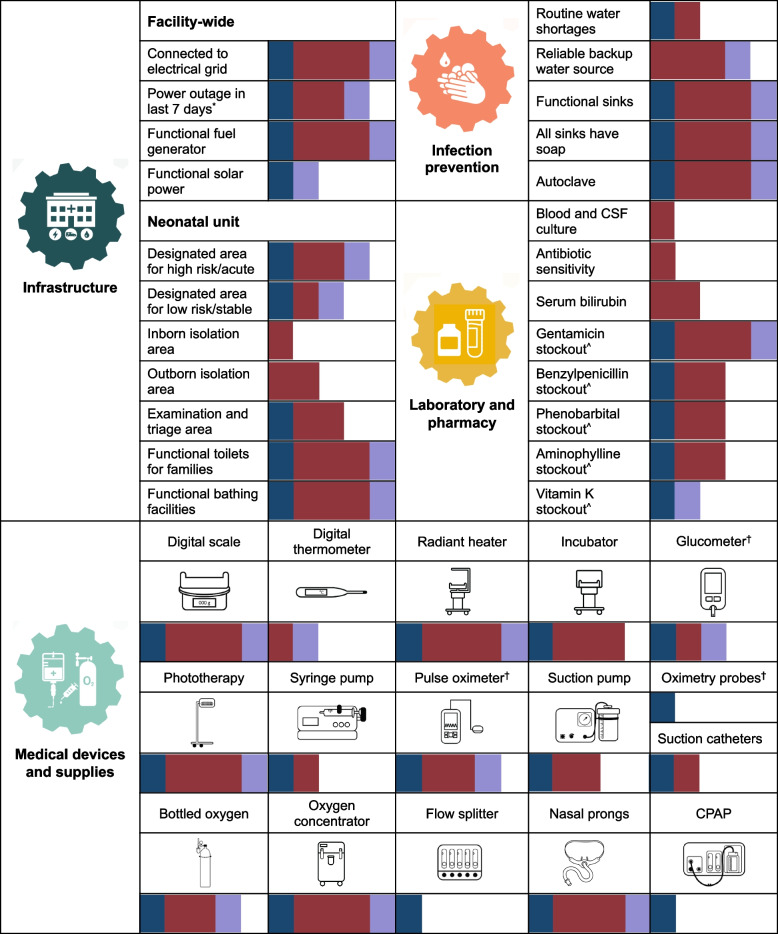
Hospital readiness to deliver neonatal care: baseline assessments after renovation of five Ugandan hospitals. Bar data indicate the number of hospitals (range: 0–5); bar colour indicates the type of facility: national referral hospital (blue); regional referral hospital (maroon); district hospital (lavender). CPAP = continuous positive airway pressure. CSF = cerebrospinal fluid. ^*^Any power outage (from grid or backup source) more than 30 min in the last 7 days. ^^^Any stockout of pharmaceutical product in the last 3 months. ^†^Data missing for Hospital-3, a regional referral facility. Images depicted in figure taken from ‘Implementation Toolkit: Small and sick newborn care’ (2022) [32], and ‘NEST360 Health Facility Assessment Summary Feedback Report’ (unpublished observations; Rebecca Penzias, Christine Bohne, Joy Lawn)

We present key findings of the HFAs, which were conducted following the necessary improvements, organised by WHO health system building block (Fig. 4). All hospitals were connected to the electrical grid and four of the five had experienced at least one power outage in the preceding 7 days (see Supplementary Table 3, Additional file 2). All hospitals had a functional fuel-operated generator for backup power but only two had solar power. Across the five neonatal units, four had an area for high-risk babies, three had an area for stable babies, one had an isolation area for babies born in the hospital, two had an isolation area for babies born outside the hospital, and three had an area for examination and triage of newly admitted babies. Routine water shortages were uncommon, and most neonatal units had a reliable backup water source. All units had functional sinks with soap, and all hospitals had a functional autoclave. Laboratory capacity was limited (see Supplementary Table 4, Additional file 2), with few hospitals able to perform blood and cerebrospinal fluid cultures (*n* = 1), antibiotic sensitivities (*n* = 1), and serum bilirubin testing (*n* = 2) on site. There was wide variability in the availability of pharmaceutical products (see Supplementary Table 5, Additional file 2). All units reported stockouts of essential medications for newborn special care (e.g., gentamicin, phenobarbital) and two reported stockouts of Vitamin K, a component of routine newborn care (WHO level-1) [8], in the preceding 3 months. Wide variability was also observed for medical devices and supplies (see Supplementary Table 6, Additional file 2). All neonatal units had functional radiant heaters (median: 2, IQR: 1–4), phototherapy units (median: 2, IQR: 0), and oxygen concentrators (median: 2, IQR: 0), as well as a digital weighing scale (mean: 1, SD: 0) and nasal prongs. Functional incubators (median: 1, IQR: 1–10), oxygen cylinders (median: 3, IQR: 2–4), and pulse oximeters (median: 1, IQR: 1–4) were available in four units, and electric suction pumps (median: 1, IQR: 1–2) and glucometers were available in three units. Syringe pumps (median: 0, IQR: 0–1), digital thermometers (mean: 1, SD: 0), and suction catheters were available in two units. Functional CPAP flow drivers (*n* = 5), flow splitter (*n* = 1), and pulse oximetry probes (*n* = 6) were only available at the national referral hospital.

## Discussion

These five Ugandan health facilities, which included a national referral hospital, a district hospital, and three regional referral hospitals, all required substantial physical and human resource inputs to allow safe implementation of iKMC. Our findings raise doubts about the affordability of rapid, widespread scale-up of iKMC in LMIC settings. We found substantial variation in the cost of improvements per hospital, per neonatal admission, and per neonatal bed. Costs were lowest at the national referral hospital, which had been recently constructed. Given the range across these facilities, largely driven by the previously available infrastructure, it would not be appropriate to extrapolate to other hospitals based on simple averages or costs per bed from our study. A standardised 20-bed neonatal unit in Uganda could be expected to offer a level of care broadly comparable to the four district and regional referral hospitals in this study, which were of similar size (range: 14–30 beds), but not to that of the national referral hospital (*n* = 106 beds). Necessary improvements to hospitals comparable to a 20-bed neonatal unit cost from $68,330 to $95,796 (financial), or $99,430 to $113,881 (economic), with costs highest where a new unit needed to be constructed. Key cost drivers were the value of donated time and floor space, infrastructure improvements, and planning and design. A single team managed the setup process at the five hospitals, suggesting that it might be possible to achieve economies of scale or lower costs if this were implemented in a larger number of hospitals. Floor space per bed and per baby in the renovated neonatal units were lowest at the national referral hospital, probably because of the higher number of beds and admissions relative to the lower-level facilities. The HFAs demonstrated broad variability in laboratory and pharmacy capacity as well as the availability of essential equipment and supplies for newborn care, even after these improvements.

To our knowledge, this study is the first to evaluate the process and costs of implementation readiness for iKMC in health facilities. Previous studies in Brazil, Colombia, Ethiopia, Indonesia, Mexico, Nicaragua, and the United Kingdom have consistently found that KMC provision among stable neonates resulted in cost savings for the hospital or health provider [33–38]. However, none of these studies considered the costs of necessary infrastructure improvements or clinical equipment, nor specifically evaluated KMC initiated before stabilisation. The favourable results of the Immediate KMC Study stimulated global demand and led to updated WHO guidelines recommending iKMC from the time of birth [17, 20], which makes it important to examine the financial and economic costs of implementing this intervention. For the five Ugandan hospitals involved in our study, the economic implications of constructing a new neonatal unit or adapting existing units to accommodate adult beds for KMC were substantial.

Two recent studies of KMC implementation among stabilised babies also identified gaps in health facility readiness. In Bangladesh, a study at eight government health facilities found that infrastructure challenges, including unavailability of adjustable beds and toilets for caregivers, were common and none of the sites had all equipment necessary to provide high-quality KMC [39]. A study across seven sites in Ethiopia and India reported that 60% of eligible infants received KMC with ≥ 8 h of skin-to-skin contact and exclusive breastfeeding in the 24 h preceding discharge, and that this coverage was achieved with government engagement and financial resources to establish and maintain KMC units with supportive policies for mothers, including beds, food, bathing, and toilets [40]. In contrast, the WHO trial and a related quality improvement study reported a median daily duration of skin-to-skin contact of 17 h among babies who received iKMC, following the establishment of mother-neonatal intensive care units with adult beds for KMC in six tertiary-level hospitals in Ghana, India, Malawi, Nigeria, and Tanzania [17, 41], illustrating the importance of providing infrastructure and advanced newborn care and equipment, including CPAP.

The findings of this study should inform planning and budgeting for the setup of safe iKMC in LMIC settings, as well as decisions regarding where and whether to implement iKMC at all. This contribution is important because the vast majority of babies in sub-Saharan Africa and South Asia lack access to hospitals with neonatal intensive care [19]. Although our precise cost estimates are specific to the five Ugandan hospitals in our study, our findings regarding the many types of improvements required, and their very substantial associated costs, especially at lower-level facilities, are likely to be relevant to and generalisable across public hospitals offering similar levels of newborn care in sub-Saharan Africa and elsewhere. Development of harmonised guidelines on the setup and implementation of iKMC, which incorporate estimated financing requirements by level of care across different geographic regions, should be a priority for policymakers [42].

This study had strengths and limitations. The data are comparable across five facilities of varying service levels and sizes. Cost data were based on detailed activity records but were collected retrospectively. Data on staff time spent away from routine duties during planning, design, and training, which accounted for around one-third of additional economic costs, could have been subject to recall bias or misreporting. We included planning and design costs for all those who attended meetings and other activities, implying these costs could be lower if fewer people were involved. The HFA modules included in this evaluation assessed readiness of facilities to provide small and sick newborn care, with a focus on physical infrastructure and clinical equipment and supplies; we did not evaluate other health system building blocks included in the HFA tool, such as human resources, information systems, and administration and management, which are also imperative for the sustainable provision of high-quality care. Finally, some HFA data, particularly pharmaceutical products, had a considerable proportion of missingness, although we note that there are few pharmaceutical products necessary for WHO level-2 care.

Policy decisions should be informed by thoughtful consideration of the level of neonatal care already available, including existing infrastructure, as well as the impact, cost-effectiveness, affordability, and sustainability of iKMC, and how these factors – and appropriate policy recommendations – may vary across settings. In a smaller trial in The Gambia, a before and after comparison showed a halving of neonatal mortality associated with infrastructure investments and improved quality of care; however, adding iKMC did not confer a significant mortality reduction [18]. Redesigning health systems in LMICs to achieve high-quality, equitable care for small and sick newborns will require investing in neonatal units with adequate space, equipment, supplies, and specialised staff [43]. Several African countries are developing standardised floor plans for neonatal units; for example, Tanzania has a national policy for a 40-bed neonatal unit in district hospitals, with 10 beds for level-2 care including CPAP [44]. In Tanzania, the cost of building a new neonatal unit is closer to $1 million [32]. Going forward with the investment case for newborn care, further research is warranted to assess the incremental neonatal unit floor space needed for the addition of iKMC.

## Conclusion

The five Ugandan hospitals in the OMWaNA trial required substantial inputs, notably for infrastructure improvements, to allow safe implementation of iKMC, highlighting the need for dedicated funding to adopt this intervention, especially in facilities that cannot afford basic equipment such as incubators. However, it also raises questions about the affordability and cost-effectiveness of recommending widespread scale-up of iKMC across LMICs. These findings should help inform planning and budgeting as well as decisions about if, where, and how to implement iKMC, particularly in LMIC settings where space, devices, and specialised staff for inpatient newborn care are often unavailable. The impact of higher quality care for small and sick newborns is expected to be substantial, so even with high set-up costs, these investments may prove cost-effective. More context-specific evidence is needed to inform policymakers, especially regarding the incremental cost-effectiveness of iKMC added to high-quality, level-2 newborn care.

## Supplementary Information

**Additional file 1.** StaRI checklist.

**Additional file 2:** Supplementary Tables.

**Additional file 3.** Neonatal unit floor plans pre- and post-renovation at the five hospitals in Uganda.

**Additional file 4.** Author reflexivity statement for equity in global health research.

## Data Availability

The datasets supporting the conclusions of this article are included within the article and its additional files.

## Abbreviations

CPAP: Continuous positive airway pressure
g: Grams
HFA: Health facility assessment
iKMC: Immediate kangaroo mother care
IQR: Interquartile range
IV: Intravenous
KMC: Kangaroo mother care
LMIC: Low- and middle-income country
LSHTM: London School of Hygiene & Tropical Medicine
m^2^: Metres squared
MRC/UVRI: Medical Research Council/Uganda Virus Research Institute
NEST360: Newborn Essential Solutions and Technologies
REDCap: Research Electronic Data Capture
SD: Standard deviation
StaRI: Standards for Reporting Implementation Studies
UGX: Ugandan Shillings
UNICEF: United Nations Children’s Fund
USD: United States Dollars
WHO: World Health Organisation
