# Supplementary material for: Process and costs for readiness to safely implement immediate kangaroo mother care: a mixed methods evaluation from the OMWaNA trial at five hospitals in Uganda
Source: BMC Health Serv Res. 2023 Jun 10;23:613. doi: 10.1186/s12913-023-09624-z (PMC10257176; doi:10.1186/s12913-023-09624-z)
Supplement: Supplementary file 2 — Additional file 2: Supplementary Tables. [file 12913_2023_9624_MOESM2_ESM.docx]

**Supplementary Tables**

Table of Contents

[Supplementary Table 1. Financial and economic costs of resources and infrastructure improvements to prepare for immediate KMC (UGX, 2020) 2](#_Toc133252471)

[Supplementary Table 2. Quantities of resources provided to hospitals to prepare for immediate KMC 4](#_Toc133252472)

[Supplementary Table 3. Baseline health facility assessment: facility and neonatal unit infrastructure 5](#_Toc133252473)

[Supplementary Table 4. Baseline health facility assessment: laboratory supplies and blood products 8](#_Toc133252474)

[Supplementary Table 5. Baseline health facility assessment: pharmaceutical products 10](#_Toc133252475)

[Supplementary Table 6. Baseline health facility assessment: medical devices and supplies 12](#_Toc133252476)

[References 15](#_Toc133252477)

# **Supplementary Table 1. Financial and economic costs of resources and infrastructure improvements to prepare for immediate KMC (UGX, 2020)**

|  | **Life-span (years)** | **Hospital-1** | | **Hospital-2** | | **Hospital-3** | | **Hospital-4** | | **Hospital-5** | | **All Hospitals** | |
| --- | --- | --- | --- | --- | --- | --- | --- | --- | --- | --- | --- | --- | --- |
|  |  | **Total cost** | **Annualised cost** | **Total cost** | **Annualised cost** | **Total cost** | **Annualised cost** | **Total cost** | **Annualised cost** | **Total cost** | **Annualised cost** | **Total cost** | **Annualised cost** |
| ***Financial costs*** |  |  |  |  |  |  |  |  |  |  |  |  |  |
| **Planning and design** | | | | | | | | | | | | | |
| Tendering and contracting | 20 | 44,500,302 | 2,992,902 | 44,500,302 | 2,992,902 | 79,246,365 | 5,326,783 | 51,815,071 | 3,484,437 | 79,246,365 | 5,326,783 | 299,308,405 | 20,123,807 |
| Scoping of work | 20 | 18,263,256 | 1,227,017 | 18,263,256 | 1,227,017 | 3,659,205 | 247,588 | 3,659,205 | 247,588 | 3,659,205 | 247,588 | 47,504,127 | 3,196,798 |
| Design | 20 | 1,827,782 | 123,794 | 1,827,782 | 123,794 | 3,047,517 | 203,896 | 1,827,782 | 123,794 | 3,047,517 | 203,896 | 11,578,380 | 779,174 |
| Site survey and inspection | 20 | 5,486,987 | 367,741 | 5,486,987 | 367,741 | 5,486,987 | 367,741 | 5,486,987 | 367,741 | 5,486,987 | 367,741 | 27,434,935 | 1,838,705 |
| Meetings | 20 | 32,918,281 | 2,213,728 | 32,918,281 | 2,213,728 | 40,233,050 | 2,705,263 | 32,918,281 | 2,213,728 | 40,233,050 | 2,705,263 | 179,220,943 | 12,051,710 |
| Transportation | 20 | NA | NA | 11,490,996 | 771,892 | 9,353,729 | 629,893 | NA | NA | 7,748,048 | 520,663 | 28,592,773 | 1,922,448 |
| **Sub-total (planning and design)** | **NA** | **102,996,608** | **6,925,182** | **114,487,604** | **7,697,074** | **141,026,853** | **9,481,164** | **95,707,326** | **6,437,288** | **139,421,172** | **9,371,934** | **593,639,563** | **39,912,642** |
| **Infrastructure improvements** | | | | | | | | | | | | | |
| Construction of new neonatal unit | 20 | NA | NA | NA | NA | NA | NA | NA | NA | 171,290,845 | 11,512,842 | 171,290,845 | 11,512,842 |
| Extension/ remodelling of existing neonatal unit | 20 | NA | NA | NA | NA | 112,437,721 | 7,558,716 | 4,158,022 | 280,357 | NA | NA | 116,595,743 | 7,839,073 |
| Repurposing of space for neonatal unit | 20 | 104,099,831 | 6,998,002 | 107,773,600 | 7,245,590 | NA | NA | NA | NA | NA | NA | 211,873,431 | 14,243,592 |
| Rental of office space | 20 | 524,304 | 36,410 | 524,304 | 36,410 | 524,304 | 36,410 | NA | NA | 524,304 | 36,410 | 2,097,216 | 145,640 |
| **Sub-total (infrastructure improvements)** | **NA** | **104,624,135** | **7,034,412** | **108,297,904** | **7,282,000** | **112,962,025** | **7,595,126** | **4,158,022** | **280,357** | **171,815,149** | **11,549,252** | **501,857,235** | **33,741,147** |
| **Clinical equipment^*^** | | | | | | | | | | | | | |
| Adjustable KMC bed^^^ | 5 | 1,733,116 | 378,664 | 4,329,149 | 946,660 | 4,329,149 | 946,660 | 8,468,966 | 1,849,628 | 4,329,149 | 946,660 | 23,189,529 | 5,068,272 |
| Oxygen concentrator^†^ | 3 | 4,456,584 | 1,576,553 | 4,456,584 | 1,576,553 | 4,456,584 | 1,576,553 | 5,046,426 | 1,784,090 | 4,456,584 | 1,576,553 | 22,872,762 | 8,090,302 |
| Masimo Rad-8^©^ pulse oximeter | 7 | 26,306,225 | 4,223,560 | 16,442,756 | 2,639,725 | 19,730,579 | 3,167,670 | 0 | 0 | 16,442,756 | 2,639,725 | 78,922,316 | 12,670,680 |
| Masimo LNC-04 patient cable | 7 | 2,708,904 | 433,279 | 1,693,065 | 273,075 | 2,031,678 | 327,690 | 0 | 0 | 1,693,065 | 273,075 | 9,142,551 | 1,467,323 |
| Glucose meter | 3 | 109,230 | 40,051 | 109,230 | 40,051 | 109,230 | 40,051 | 0 | 0 | 109,230 | 40,051 | 436,920 | 160,204 |
| Digital baby weighing scale^‡^ | 3 | 3,120,337 | 1,103,223 | 3,120,337 | 1,103,223 | 3,109,414 | 1,099,582 | 0 | 0 | 3,120,337 | 1,103,223 | 12,470,425 | 4,409,251 |
| Neonatal measuring mat^#^ | 3 | 171,127 | 58,256 | 171,127 | 58,256 | 171,127 | 58,256 | 0 | 0 | 171,127 | 58,256 | 684,508 | 247,588 |
| Neonatal ventilation bag and mask | 3 | 83,743 | 29,128 | 83,743 | 29,128 | 83,743 | 29,128 | 83,743 | 29,128 | 0 | 0 | 334,972 | 116,512 |
| Digital axillary thermometer | 3 | 32,769 | 10,923 | 32,769 | 10,923 | 32,769 | 10,923 | 0 | 0 | 32,769 | 10,923 | 131,076 | 43,692 |
| Paediatric stethoscope | 3 | 637,175 | 225,742 | 637,175 | 225,742 | 637,175 | 225,742 | 0 | 0 | 637,175 | 225,742 | 2,548,700 | 902,968 |
| Training on KMC and clinical guidelines | 35 | 1,114,146 | 50,974 | 4,864,376 | 225,742 | 5,002,734 | 233,024 | 0 | 0 | 6,564,723 | 305,844 | 17,545,979 | 815,584 |
| Neonatal resuscitator | 5 | 83,743 | 18,205 | 83,743 | 18,205 | 83,743 | 18,205 | 83,743 | 18,205 | 0 | 0 | 334,972 | 72,820 |
| Penguin newborn suction | 3 | 36,410 | 14,564 | 36,410 | 14,564 | 36,410 | 14,564 | 36,410 | 14,564 | 0 | 0 | 145,640 | 58,256 |
| NeoNatalie^©^ manikin^§^ | 3 | 334,972 | 120,153 | 334,972 | 120,153 | 334,972 | 120,153 | 334,972 | 120,153 | 0 | 0 | 1,339,888 | 480,612 |
| PreemieNatalie^©^ manikin^§^ | 3 | 240,306 | 83,743 | 240,306 | 83,743 | 240,306 | 83,743 | 240,306 | 83,743 | 0 | 0 | 961,224 | 334,972 |
| **Sub-total (clinical equipment)** | **NA** | **41,168,787** | **8,370,659** | **37,651,581** | **7,529,588** | **40,389,613** | **7,955,585** | **14,294,566** | **3,899,511** | **37,556,915** | **7,183,693** | **171,061,462** | **34,939,036** |
| **Sub-total (financial cost)** | **NA** | **248,789,530** | **22,330,253** | **260,437,089** | **22,508,662** | **294,378,491** | **25,031,875** | **114,159,914** | **10,617,156** | **348,793,236** | **28,104,879** | **1,266,558,260** | **108,592,825** |
| ***Additional economic costs*** | | | | | | | | | | | | | |
| Planning and design^¶^ | 20 | 29,972,712 | 2,013,473 | 41,463,708 | 2,785,365 | 41,951,602 | 2,818,134 | 25,661,768 | 1,725,834 | 40,345,921 | 2,712,545 | 179,395,711 | 12,055,351 |
| Training^\|\|^ | 20 | 24,209,009 | 1,627,527 | 26,124,175 | 1,754,962 | 25,767,357 | 1,733,116 | 24,209,009 | 1,627,527 | 25,501,564 | 1,714,911 | 125,811,114 | 8,458,043 |
| Donated space^Ω^ | 20 | 59,053,379 | 3,968,690 | 48,760,272 | 3,276,900 | 0 | 0 | 0 | 0 | 0 | 0 | 107,813,651 | 7,245,590 |
| **Sub-total (additional economic costs)** | **NA** | **113,235,100** | **7,609,690** | **116,348,155** | **7,817,227** | **67,718,959** | **4,551,250** | **49,870,777** | **3,353,361** | **65,847,485** | **4,427,456** | **413,020,476** | **27,758,984** |
| ***Total economic cost*** | **NA** | **362,023,902** | **29,939,943** | **376,785,244** | **30,325,889** | **362,097,450** | **29,583,125** | **164,030,691** | **13,970,517** | **414,640,721** | **32,532,335** | **1,679,578,736** | **136,351,809** |

KMC=kangaroo mother care. NA=not applicable. UGX=Ugandan Shillings. Costs were annualised using a discount rate of 3%,^1^ and assumptions about the lifespan of capital improvements, equipment, and activities. ^*^Clinical equipment and durable goods that were essential to allow safe implementation of immediate KMC, in accordance with the OMWaNA trial protocol.^2^ ^^^E3A India Narag adjustable bed, Crown Health Care Ltd., Kampala, Uganda. ^†^USA Airsep oxygen concentrator, Crown Health Care Ltd., Kampala, Uganda. ^‡^Seca 384 digital baby weighing scale. ^#^Seca 210 neonatal measuring mat. ^§^Leardal NeoNatalie^©^ and PreemieNatalie^©^ neonatal resuscitation manikins. ^¶^Time spent by hospital staff away from normal duties to attend meetings or inspect the study sites. ^||^Time spent by the trainers away from normal duties to conduct staff training. ^Ω^Calculated as the difference between the financial cost of renovations and the economic cost to construct a new space of equivalent size, estimated using the unit financial cost of improvements at Hospital-5.

# **Supplementary Table 2.** **Quantities of resources provided to hospitals to prepare for immediate KMC**

|  | **Hospital-1** | **Hospital-2** | **Hospital-3** | **Hospital-4** | **Hospital-5** | **Total** |
| --- | --- | --- | --- | --- | --- | --- |
| ***Financial costs*** |  |  |  |  |  |  |
| **Planning and design** | **Person- hours** | **Person-hours** | **Person-hours** | **Person-hours** | **Person-hours** | **Person-hours** |
| Tendering and contracting | 138 | 240 | 138 | 240 | 156 | 912 |
| Scoping of work | 80 | 16 | 80 | 16 | 16 | 208 |
| Design | 24 | 40 | 24 | 40 | 24 | 152 |
| Site survey and inspection | 24 | 24 | 24 | 24 | 24 | 120 |
| Meetings | 120 | 144 | 120 | 144 | 120 | 648 |
| **Transport** | **No. of trips** | **No. of trips** | **No. of trips** | **No. of trips** | **No. of trips** | **No. of trips** |
|  | 11 | 21 | 21 | 13 | 20 | 86 |
| **Clinical setup^*^** | **Units** | **Units** | **Units** | **Units** | **Units** | **Units** |
| KMC adjustable bed | 2 | 5 | 5 | 10 | 5 | 27 |
| Oxygen concentrator | 1 | 1 | 1 | 1 | 1 | 5 |
| Masimo Rad-8^©^ pulse oximeter | 8 | 5 | 6 | 0 | 5 | 24 |
| Masimo LNC-04 patient cable | 8 | 5 | 6 | 0 | 5 | 24 |
| Glucose meter | 1 | 1 | 1 | 0 | 1 | 4 |
| Digital baby weighing scale | 1 | 1 | 1 | 0 | 1 | 4 |
| Neonatal measuring mat | 1 | 1 | 1 | 0 | 1 | 4 |
| Neonatal ventilation bag and mask | 1 | 1 | 1 | 1 | 0 | 4 |
| Digital axillary thermometer | 2 | 2 | 2 | 0 | 2 | 8 |
| Paediatric stethoscope | 12 | 12 | 12 | 0 | 12 | 48 |
| Training on KMC and clinical guidelines | 0 | 0 | 0 | 0 | 0 | 0 |
| Neonatal resuscitator | 1 | 1 | 1 | 1 | 0 | 4 |
| Penguin newborn suction | 2 | 2 | 2 | 2 | 0 | 8 |
| NeoNatalie^©^ manikin | 1 | 1 | 1 | 1 | 0 | 4 |
| PreemieNatalie^©^ manikin | 1 | 1 | 1 | 1 | 0 | 4 |
| ***Additional economic costs*** |  |  |  |  |  |  |
| **Planning, design, and training** | **Person-hours** | **Person-hours** | **Person-hours** | **Person-hours** | **Person-hours** | **Person-hours** |
| Planning and design | 386 | 464 | 386 | 464 | 340 | 2040 |
| Training | 120 | 120 | 120 | 120 | 120 | 600 |
| **Training** | **No. of rooms** | **No. of rooms** | **No. of rooms** | **No. of rooms** | **No. of rooms** | **No. of rooms** |
|  | 1 | 1 | 1 | 1 | 1 | 5 |
| **Repurposed, remodelled, or newly constructed space** |  |  |  |  |  |  |
| Floor space^^^, m^2^ | 94 | 34 | 99 | 38 | 99 |  |

KMC=kangaroo mother care. m^2^=metres squared. ^*^Clinical setup resources included equipment and durable goods that were essential to allow safe implementation of immediate KMC, in accordance with the OMWaNA trial protocol.^2^ ^^^This figure reflects the floor space of the area where renovations were completed.

# **Supplementary Table 3. Baseline health facility assessment: facility and neonatal unit infrastructure**

|  | **Hospital-1** | **Hospital-2** | **Hospital-3** | **Hospital-4** | **Hospital-5** |
| --- | --- | --- | --- | --- | --- |
| **Facility infrastructure** |  |  |  |  |  |
| Connected to electric grid | Yes | Yes | Yes | Yes | Yes |
| Power outage >30 min in past 7 days^*^ | Yes | Yes | Yes | Yes | No |
| Functional generator/solar power | Yes | Yes | Yes | Yes | Yes |
| Functional fuel-operated generator | Yes | Yes | Yes | Yes | Yes |
| Sufficient fuel to run generator | Yes | No | No | No | No |
| Functional battery-operated generator | No | No | No | No | Yes |
| Functional solar power | No | Yes | No | Yes | No |
| Main water source | Piped | Solar pump^^^ | Piped | Piped | Piped |
| Routine water shortages | Yes | No | No | Yes | Unknown |
| Reliable backup water supply | Yes | Yes | Yes | Yes | Yes |
| Backup water source | Tank, rainwater | Tank, hand pump^^^ | Tank, rainwater | Tank | Tank |
| Functional autoclave | Yes | Yes | Yes | Yes | Yes |
| Functional hot air steriliser/dry oven | Yes | No | No | No | Yes |
| Functional steriliser, electric | Yes | No | No | Yes | No |
| Functional steriliser, kerosene heated | Yes | No | No | No | No |
| Functional sterilisation drum/stand | Yes | No | Yes | Yes | Yes |
| Autoclave sterilisation tapes | Yes | No | No | Yes | No |
| Motor vehicle ambulances, n | 2 | Unknown | 2 | 1 | 2 |
| Out-of-service motor vehicles, n | 0 | Unknown | 1 | 0 | 1 |
| Motorcycle ambulances, n | 0 | 1 | 0 | 0 | 0 |
| Out-of-service motorcycles, n | NA | 0 | NA | NA | NA |
| Routine ambulance maintenance | No | Yes | Yes | No | Yes |
| Ambulance maintenance/repair funds | Yes | Yes | Yes | Yes | Yes |
| **Neonatal unit infrastructure** |  |  |  |  |  |
| Functional neonatal cots, n | 0 | 6 | 9 | 60 | 12 |
| Multiple babies per cot | Never | Sometimes | Sometimes | Sometimes | Frequently |
| Multiple babies per radiant warmer | Frequently | Sometimes | Frequently | Sometimes | Sometimes |
| Time from labour ward to NSCU | <5 min | <5 min | <5 min | <5 min | <5 min |
| Time from operating theatre to NSCU | <5 min | <5 min | <5 min | <5 min | <5 min |
| Neonatal triage/examination area | Yes | No | Yes | Yes | No |
| Neonatal feed/milk preparation area | No | No | No | No | No |
| Clean supply/equipment storage space | Yes | Yes | Yes | Yes | Yes |
| Functional air filtration system | Yes | No | No | No | No |
| Functional heating system | No | No | No | No | No |
| Functional room thermometer | Yes | Yes | Yes | No | No |
| Adequate lighting- daytime | Yes | Yes | Yes | Yes | Yes |
| Adequate lighting- night-time | No | No | Yes | Yes | Yes |
| Walled/piped oxygen | No | No | No | Yes | No |
| Walled/piped blended oxygen | No | No | No | Yes | No |
| Staff work area/nurses’ station | Yes | Yes | Yes | Yes | Yes |
| Staff seating | Yes | Yes | Yes | Yes | Yes |
| Private space for counselling | No | No | Yes | No | No |
| Routine water shortages | No | No | Yes | Yes | No |
| Reliable backup water source | Yes | Yes | Yes | No | Yes |
| Fire extinguisher | Yes | Yes | No | No | No |
| Maternal seating in NSCU | Yes | No | Yes | Yes | Yes |
| Maternal beds in NSCU | No | Yes | No | No | No |
| Maternal shelter | Yes | Yes | No | Yes | Yes |
| Private space for milk expression | No | No | No | No | No |
| Refrigerated breastmilk storage | No | No | No | No | No |
| Human milk bank | No | No | No | No | No |
| Waiting area for NSCU | Yes | No | Yes | Yes | Yes |
| Adequate seating in waiting area | No | NA | No | No | No |
| Visiting times for NSCU^†^ | Specific | Specific | Specific | Specific | Specific |
| Adequate space in KMC unit | Yes | No | No | No | Yes |
| Total beds in KMC unit, n | 9 | 5 | 5 | 0 | 5 |
| Adjustable beds in KMC unit, n | 5 | 5 | 4 | 0 | 1 |
| KMC reclining chairs, n | 0 | 0 | 0 | 0 | 0 |
| Handwashing area | Yes | Yes | Yes | Yes | Yes |
| All sinks functional | Yes | Yes | Yes | Yes | Yes |
| All sinks have soap | Yes | Yes | Yes | Yes | Yes |
| Staff handwashing | Always | Always | Always | Always | Sometimes |
| Visitor handwashing | Sometimes | Sometimes | Always | Always | Sometimes |
| Storage area for shoes | Yes | No | Yes | Yes | Yes |
| Staff/visitor shoe removal before entry | Always | Always | Always | Always | Always |
| Functional toilets for staff | Yes | Yes | Yes | Yes | Yes |
| Time from NSCU to staff toilets | 1 min | 3 min | 2 min | 1 min | 1 min |
| Functional toilets for mothers | Yes | Yes | Yes | Yes | Yes |
| Time from NSCU to maternal toilets | 1 min | 2 min | 5 min | 3 min | 2 min |
| Bathing area for mothers | Yes | Yes | Yes | Yes | Yes |
| Cooking area/equipment for mothers | Yes | Yes | Yes | No | No |
| Laundry area for mothers | No | No | Yes | Yes | No |
| Supply/equipment cleaning area | No | Yes | Yes | Yes | Yes |
| Supply/equipment sterilisation area | No | Yes | No | No | No |
| Functioning incinerator | Yes | Yes | Yes | No | No |
| Storage space for soiled utility | Yes | Yes | No | Yes | Yes |
| Trash collection at least once daily | Yes | Yes | Yes | Yes | Yes |
| Liquid spills/trash on floor observed | No | No | No | Yes | Yes |
| Any vermin observed | Yes | Yes | Yes | No | Yes |
| Generator/solar powers neonatal unit | Yes | Yes | No | Yes | No |
| Generator/solar power exclusively covers neonatal unit | No | Yes | No | Yes | No |
| Generator/solar powers all neonatal unit lighting | Yes | Yes | No | Yes | No |
| Generator/solar powers all neonatal devices and equipment | Yes | Yes | No | Yes | No |
| Sufficient electrical outlets for all neonatal devices and equipment | Yes | Yes | No | Yes | Yes |
| Electrical outlets, n | 10 | 34 | 21 | 224 | Unknown |
| Power strips/surge protectors, n | 0 | 2 | 2 | 3 | 2 |
| Voltage stabiliser | Yes | No | No | No | Yes |
| Any broken equipment due to electricity problems in past year | No | Yes | No | Unknown | Yes |
| Adequate power supply amperage for neonatal devices/equipment | Yes | Yes | No | Yes | Yes |
| Fuel available for neonatal transfer | Unknown | No | No | No | No |
| Driver available for neonatal transfer | Yes | Yes | Yes | Yes | Yes |
| Nurse available for neonatal transfer | Unknown | No | No | Yes | Yes |
| Distance to nearest referral facility | 73 km | 40 km | 80 km | 4 km | 75 km |
| Drive time to nearest referral facility | 120 min | 45 min | 126 min | 10 min | 78 min |

KMC=kangaroo mother care. km=kilometre. min=minute. NA=not applicable. NSCU=newborn special care unit. ^*^Grid or backup power outage. ^^^Borehole solar and hand pumps. ^†^Mothers and caregivers allowed to visit neonatal unit at specific times only.

# **Supplementary Table 4. Baseline health facility assessment: laboratory supplies and blood products**

|  | **Hospital-1** | **Hospital-2** | **Hospital-3** | **Hospital-4** | **Hospital-5** |
| --- | --- | --- | --- | --- | --- |
| **Laboratory guidelines** |  |  |  |  |  |
| Biosafety guidelines | Yes | Yes | Yes | No | Yes |
| Quality control guidelines | Yes | Yes | Yes | No | Yes |
| Standard operating procedures | Yes | Yes | Yes | Yes | Yes |
| **Laboratory supplies and reagents** |  |  |  |  |  |
| Albumin (bovine) | Unavailable | Unavailable | Inadequate | Adequate | Unknown |
| Anticoagulant bottles | Unavailable | Unavailable | Unavailable | Adequate | Unavailable |
| Artery forceps | Unavailable | Unavailable | Unavailable | Unavailable | Unavailable |
| Beakers | Inadequate | Inadequate | Inadequate | Inadequate | Inadequate |
| Blood collection bags and needles | Adequate | Unavailable | Unavailable | Inadequate | Adequate |
| Blood lancets | Adequate | Adequate | Adequate | Adequate | Adequate |
| Blood typing reagents | Adequate | Inadequate | Adequate | Adequate | Adequate |
| Bowls | Unavailable | Unavailable | Inadequate | Inadequate | Adequate |
| Buffered water | Unavailable | Unavailable | Unavailable | Unavailable | Unavailable |
| Capillary tubes | Unavailable | Adequate | Unavailable | Unavailable | Unavailable |
| CD4 machine | Adequate | Adequate | Adequate | Adequate | Adequate |
| Cotton wool | Adequate | Inadequate | Adequate | Adequate | Adequate |
| Cross-matching reagents | Adequate | Inadequate | Adequate | Adequate | Adequate |
| Cover slips | Adequate | Inadequate | Adequate | Inadequate | Adequate |
| Ethanol | Unavailable | Inadequate | Inadequate | Inadequate | Adequate |
| Functional balance, n | 2 | 0 | 1 | 0 | 0 |
| Functional blood bank refrigerator, n | 2 | 3 | 1 | 1 | 1 |
| Functional blood collection scale, n | 0 | 0 | 0 | 0 | 1 |
| Functional centrifuge (electric), n | 2 | 2 | 3 | 5 | 1 |
| Functional counting chambers, n | 1 | 0 | 0 | 0 | 1 |
| Functional haemoglobinometer, n | 1 | 0 | 0 | 1 | 2 |
| Functional laboratory refrigerator, n | 2 | 2 | 3 | 7 | 2 |
| Functional compound microscope, n | 3 | 3 | 1 | 1 | 3 |
| Functional sinks, n | 18 | 9 | 4 | 8 | 8 |
| Functional spectrophotometer, n | 2 | 0 | 0 | 0 | 2 |
| Functional tally counter, n | 1 | 0 | 1 | 0 | 1 |
| Functional timer or clock, n | 1 | 1 | 2 | 0 | 0 |
| Functional water bath (37° C), n | 1 | 0 | 0 | 2 | 0 |
| Funnel | Inadequate | Inadequate | Inadequate | Unavailable | Inadequate |
| Giemsa stain | Unavailable | Unavailable | Unavailable | Unavailable | Unavailable |
| Glass rods | Unavailable | Unavailable | Adequate | Adequate | Inadequate |
| Hydrochloric acid | Adequate | Adequate | Adequate | Unavailable | Adequate |
| Immersion oil | Unavailable | Adequate | Adequate | Adequate | Unavailable |
| Lugols iodine solution | Unavailable | Inadequate | Inadequate | Adequate | Unavailable |
| Measuring cylinder | Adequate | Adequate | Inadequate | Adequate | Inadequate |
| Methanol | Unavailable | Inadequate | Unavailable | Inadequate | Unavailable |
| Microbiology slides | Adequate | Adequate | Adequate | Adequate | Adequate |
| Petri dishes | Unavailable | Unavailable | Adequate | Inadequate | Unavailable |
| Pipette (5 mL) | Inadequate | Unavailable | Inadequate | Inadequate | Inadequate |
| Pipette (graduated) | Unavailable | Inadequate | Inadequate | Inadequate | Unavailable |
| Pipette (dropping) | Unavailable | Inadequate | Inadequate | Adequate | Adequate |
| Rack for drying slides | Adequate | Inadequate | Inadequate | Unavailable | Adequate |
| Test tubes | Inadequate | Inadequate | Inadequate | Adequate | Inadequate |
| Test tube rack | Adequate | Adequate | Adequate | Adequate | Adequate |
| Wash bottle | Unavailable | Unavailable | Unavailable | Inadequate | Unavailable |
| Wright stain | Unavailable | Unavailable | Unavailable | Unavailable | Unavailable |
| **Laboratory tests** |  |  |  |  |  |
| Albumin | Available | Outsourced | Not today | Unavailable | Available |
| Antibiotic sensitivity | Unavailable | Unavailable | Available | Unavailable | Unavailable |
| Bilirubin (conjugated) | Available | Outsourced | Not today | Unavailable | Available |
| Bilirubin (unconjugated) | Available | Unavailable | Not today | Unavailable | Available |
| Blood gas analysis | Unavailable | Unavailable | Unavailable | Unavailable | Unavailable |
| Blood typing | Available | Not today | Available | Available | Available |
| Calcium | Unavailable | Unavailable | Not today | Unavailable | Available |
| Coagulation profile | Unavailable | Not today | Unavailable | Unavailable | Unavailable |
| Complete blood count | Available | Not today | Not today | Available | Available |
| Coombs’s test | Unavailable | Not today | Not today | Available | Unavailable |
| C-reactive protein | Unavailable | Unavailable | Unavailable | Unavailable | Unavailable |
| Creatinine | Available | Not today | Not today | Unavailable | Available |
| CSF cell count | Unavailable | Unavailable | Unavailable | Unavailable | Unavailable |
| CSF fluid analysis (glucose, protein) | Unavailable | Unavailable | Not today | Unavailable | Unavailable |
| Culture (blood, CSF, pus, urine) | Unavailable | Unavailable | Available | Unavailable | Unavailable |
| Sensitivity (blood, CSF, pus, urine) | Unavailable | Unavailable | Available | Unavailable | Unavailable |
| Electrolytes | Available | Available | Not today | Unavailable | Available |
| Glucose | Not today | Not today | Not today | Available | Not today |
| Glucose-6-phosphate dehydrogenase | Unavailable | Unavailable | Unavailable | Unavailable | Unavailable |
| Gram staining | Available | Not today | Available | Unavailable | Available |
| Haemoglobin | Available | Not today | Not today | Available | Available |
| Hepatitis B screening | Not today | Available | Available | Unavailable | Available |
| Hepatitis C screening | Unavailable | Unavailable | Unavailable | Unavailable | Unavailable |
| HIV screening | Available | Available | Not today | Unavailable | Available |
| Liver function tests | Available | Not today | Not today | Unavailable | Available |
| Magnesium | Not today | Outsourced | Not today | Unavailable | Unavailable |
| Malaria screening | Available | Available | Available | Unavailable | Available |
| Malaria test^*^ | Available | Available | Unknown | Available | Unknown |
| Stool analysis | Available | Available | Available | Available | Available |
| Syphilis screening | Available | Not today | Available | Unavailable | Available |
| Tuberculosis (skin) | Unavailable | Unavailable | Unavailable | Unavailable | Unavailable |
| Tuberculosis (staining) | Available | Available | Available | Unavailable | Available |
| Tuberculosis (GeneXpert) | Available | Available | Available | Unavailable | Available |
| Urea | Available | Not today | Not today | Unavailable | Available |
| Urinalysis | Available | Available | Available | Available | Available |
| **Blood products** |  |  |  |  |  |
| Fresh frozen plasma (stored) | Unavailable | Unavailable | Not today | Available | Unavailable |
| Fresh frozen platelets (stored) | Unavailable | Unavailable | Not today | Available | Unavailable |
| Fresh whole blood (stored) | Available | Available | Not today | Available | Unavailable |
| Packed red blood cells (stored) | Available | Available | Not today | Available | Available |
| Rh-negative blood (stored) | Available | Not today | Unavailable | Available | Available |

CSF=cerebrospinal fluid. HIV=human immunodeficiency virus. ^*^Blood film microscopy or rapid diagnostic test.

# **Supplementary Table 5. Baseline health facility assessment: pharmaceutical products**

|  | **Hospital-1** | **Hospital-2** | **Hospital-3** | **Hospital-4** | **Hospital-5** |
| --- | --- | --- | --- | --- | --- |
| **Antibiotics** |  |  |  |  |  |
| Amoxicillin (oral suspension) | Unavailable | Inadequate | Unavailable | Adequate | Unavailable |
| Amoxicillin (injection) | Unavailable | Unavailable | Unavailable | Unavailable | Unavailable |
| Amikacin (IV/IM) | Inadequate | Unavailable | Unavailable | Inadequate | Unknown |
| Ampicillin (IV/IM) | Adequate | Inadequate | Inadequate | Adequate | Adequate |
| Ampicillin (oral) | Unavailable | Inadequate | Unknown | Unavailable | Inadequate |
| Benzathine benzylpenicillin (IM) | Adequate | Adequate | Unknown | Unavailable | Adequate |
| Benzylpenicillin (IV/IM) | Adequate | Adequate | Unknown | Unavailable | Adequate |
| Cefotaxime (IV/IM) | Unavailable | Unavailable | Unknown | Unavailable | Adequate |
| Ceftriaxone (IV/IM) | Inadequate | Inadequate | Unknown | Unavailable | Adequate |
| Ethambutol (oral) | Adequate | Adequate | Unknown | Adequate | Adequate |
| Flucloxacillin (IV/IM) | Unavailable | Unavailable | Unknown | Unavailable | Unavailable |
| Flucloxacillin (oral) | Unavailable | Unavailable | Unknown | Unavailable | Unavailable |
| Gentamicin (IV/IM) | Unavailable | Unavailable | Unknown | Unavailable | Unavailable |
| Isoniazid (oral) | Adequate | Adequate | Unknown | Adequate | Adequate |
| Meropenem (IV) | Unavailable | Unavailable | Unknown | Unavailable | Unavailable |
| Metronidazole (IV) | Inadequate | Adequate | Unknown | Unavailable | Adequate |
| Metronidazole (oral) | Adequate | Adequate | Unknown | Adequate | Adequate |
| Moxifloxacin eye drops | Unavailable | Unavailable | Unknown | Unavailable | Unavailable |
| Piperazine (oral) | Unavailable | Unavailable | Unknown | Unavailable | Unavailable |
| Procaine benzylpenicillin (IM) | Adequate | Unavailable | Unknown | Unavailable | Unavailable |
| Tetracycline 1% eye ointment | Adequate | Inadequate | Unknown | Adequate | Adequate |
| Vancomycin (IV) | Unavailable | Unavailable | Unknown | Unavailable | Unavailable |
| **Analgesics and local anaesthetics** |  |  |  |  |  |
| Lidocaine solution | Adequate | Inadequate | Unknown | Adequate | Inadequate |
| Morphine (IV) | Unavailable | Unavailable | Inadequate | Unavailable | Unknown |
| Morphine (oral) | Inadequate | Adequate | Adequate | Adequate | Unknown |
| Paracetamol (oral) | Adequate | Adequate | Adequate | Adequate | Adequate |
| Paracetamol (rectal) | Inadequate | Inadequate | Inadequate | Adequate | Adequate |
| Paracetamol (injection) | Unavailable | Unavailable | Unavailable | Unavailable | Unknown |
| Proparacaine 0.5% eye drops | Unavailable | Unavailable | Unknown | Unavailable | Unavailable |
| **Anticonvulsants** |  |  |  |  |  |
| Paraldehyde (rectal) | Unavailable | Unavailable | Unavailable | Unavailable | Unavailable |
| Phenobarbital (IV/IM) | Inadequate | Inadequate | Unavailable | Adequate | Adequate |
| Phenobarbital (oral) | Adequate | Adequate | Adequate | Unavailable | Adequate |
| Phenytoin (IV) | Inadequate | Adequate | Unavailable | Unavailable | Adequate |
| **Antifungals and antivirals** |  |  |  |  |  |
| Acyclovir (IV) | Unavailable | Unavailable | Unavailable | Unavailable | Unavailable |
| Acyclovir 3% topical eye ointment | Unavailable | Unavailable | Unavailable | Unavailable | Unavailable |
| Azidothymidine/zidovudine (oral) | Inadequate | Adequate | Unavailable | Unavailable | Adequate |
| Fluconazole (IV) | Inadequate | Inadequate | Unknown | Unavailable | Unavailable |
| Fluconazole (oral) | Inadequate | Inadequate | Unknown | Unavailable | Adequate |
| Miconazole cream | Inadequate | Unavailable | Unknown | Unavailable | Unavailable |
| Nevirapine (oral) | Adequate | Adequate | Adequate | Adequate | Inadequate |
| Nystatin (oral) | Inadequate | Inadequate | Unknown | Unavailable | Inadequate |
| Nystatin cream | Inadequate | Inadequate | Unknown | Unavailable | Inadequate |
|  |  |  |  |  |  |
| **Emergency drugs** |  |  |  |  |  |
| Epinephrine (IV) | Adequate | Inadequate | Inadequate | Adequate | Inadequate |
| Calcium gluconate 10% (injection) | Adequate | Adequate | Unavailable | Unavailable | Unavailable |
| Dexamethasone (IM) | Adequate | Adequate | Adequate | Adequate | Inadequate |
| Hydrocortisone (injection) | Adequate | Inadequate | Unavailable | Adequate | Adequate |
| Naloxone (IV) | Unavailable | Adequate | Unavailable | Inadequate | Unavailable |
| **IV fluids** |  |  |  |  |  |
| Dextrose 10% with normal saline | Adequate | Inadequate | Inadequate | Unavailable | Adequate |
| Dextrose 5% with normal saline | Adequate | Inadequate | Adequate | Unavailable | Adequate |
| Dextrose/glucose 10% | Adequate | Inadequate | Adequate | Adequate | Adequate |
| Potassium chloride 7.5%, 10%, 15% | Unavailable | Unavailable | Unavailable | Unavailable | Unknown |
| Ringer’s lactate | Adequate | Adequate | Unavailable | Adequate | Adequate |
| Sodium chloride 0.9% | Adequate | Adequate | Adequate | Adequate | Adequate |
| Water for injection | Adequate | Adequate | Unavailable | Unavailable | Adequate |
| **Vaccines and immune therapies** |  |  |  |  |  |
| Anti-Rho immunoglobulin (IM/IV) | Unavailable | Unavailable | Inadequate | Unavailable | Unavailable |
| BCG vaccine | Adequate | Adequate | Adequate | Adequate | Adequate |
| Hepatitis B immunoglobulin | Unavailable | Unavailable | Unknown | Unavailable | Unavailable |
| Hepatitis B vaccine | Adequate | Adequate | Adequate | Unavailable | Adequate |
| Poliomyelitis vaccine (oral) | Adequate | Adequate | Adequate | Adequate | Adequate |
| Tetanus immunoglobulin (IM) | Unavailable | Adequate | Adequate | Adequate | Adequate |
| **Vitamins and supplements** |  |  |  |  |  |
| Ferrous fumarate (oral) | Unavailable | Unavailable | Unknown | Adequate | Unavailable |
| Folic acid | Adequate | Inadequate | Unknown | Adequate | Adequate |
| Human milk fortifier | Unavailable | Unavailable | Unknown | Unavailable | Unavailable |
| Multivitamin (oral) | Adequate | Adequate | Unknown | Adequate | Adequate |
| Oral rehydration solution | Adequate | Inadequate | Unknown | Unavailable | Adequate |
| Phosphate solution | Unavailable | Unavailable | Unknown | Unavailable | Unavailable |
| Vitamin K (IV/IM) | Adequate | Inadequate | Unknown | Unavailable | Adequate |
| **Other drugs** |  |  |  |  |  |
| Aminophylline (IV/oral) | Inadequate | Adequate | Adequate | Unavailable | Adequate |
| Artificial tears | Unavailable | Unavailable | Unknown | Unavailable | Unavailable |
| Caffeine (IV) | Unavailable | Unavailable | Unknown | Unavailable | Unavailable |
| Caffeine (oral) | Unavailable | Unavailable | Unavailable | Unavailable | Unavailable |
| Chlorhexidine digluconate 7.1% gel | Unavailable | Unavailable | Unknown | Adequate | Unknown |
| Dilating eye drops^*^ | Unavailable | Unavailable | Unknown | Unavailable | Unavailable |
| Furosemide (IV) | Adequate | Adequate | Unknown | Adequate | Adequate |
| Furosemide (oral) | Adequate | Adequate | Unknown | Unavailable | Adequate |
| Glycerine chip (rectal) | Unavailable | Unavailable | Unknown | Unavailable | Unavailable |
| Sucrose 30% (oral) | Unavailable | Unavailable | Unknown | Adequate | Unavailable |
| Surfactant | Unavailable | Unavailable | Unknown | Inadequate | Unavailable |
| Water-based lubricant | Inadequate | Adequate | Unknown | Unavailable | Inadequate |
| Zinc oxide cream | Unavailable | Unavailable | Unknown | Adequate | Unavailable |

BCG=Bacillus Calmette–Guérin vaccine for prevention of tuberculosis. IM=intramuscular. IV=intravenous. ^*^Tropicamide 0.5% phenylephrine 2.5% ocular drops for dilation.

# **Supplementary Table 6. Baseline health facility assessment: medical devices and supplies**

|  | **Hospital-1** | **Hospital-2** | **Hospital-3** | **Hospital-4** | **Hospital-5** |
| --- | --- | --- | --- | --- | --- |
| **Neonatal care** |  |  |  |  |  |
| Baby diapers (cloth or disposable) | Unavailable | Unavailable | Unavailable | Unavailable | Unavailable |
| Blood collection needles (22G) | Unavailable | Unavailable | Unavailable | Inadequate | Unavailable |
| Blood collection tubes (small size) | Unavailable | Unavailable | Unavailable | Adequate | Unavailable |
| Blood collection tube holders | Unavailable | Unavailable | Unavailable | Adequate | Adequate |
| Blood pressure apparatus | Unavailable | Unavailable | Unavailable | Inadequate | Unavailable |
| Blood pressure cuff (neonatal size) | Unavailable | Unavailable | Unavailable | Inadequate | Unavailable |
| Capillary sample tubes (glass) | Adequate | Unavailable | Unavailable | Unavailable | Unavailable |
| Clean blankets or towels for drying | Adequate | Unavailable | Unavailable | Unavailable | Unavailable |
| Dressing/procedure trays (sterile) | Adequate | Unavailable | Unavailable | Adequate | Unavailable |
| Dressing/procedure trolley | Unavailable | Unavailable | Inadequate | Adequate | Unavailable |
| Blood glucose meter | Unavailable | Inadequate | Unknown | Adequate | Adequate |
| Blood glucose test strips | Inadequate | Inadequate | Inadequate | Adequate | Inadequate |
| Echocardiography device | Inadequate | Unavailable | Unknown | Unavailable | Unavailable |
| ECG recorder | Inadequate | Unavailable | Unknown | Unavailable | Unavailable |
| ECG leads (consumable) | Inadequate | Unavailable | Unknown | Unavailable | Unavailable |
| ECG leads (reusable) | Inadequate | Unavailable | Unknown | Unavailable | Unavailable |
| Flashlights/torches | Unavailable | Unavailable | Unknown | Adequate | Unavailable |
| Functional patient monitor^*^, n | 0 | 0 | 0 | 3 | 0 |
| Functional pulse oximeter, n | 1 | 1 | Unknown | 6 | 1 |
| Pulse oximetry probes (consumable) | Unavailable | Unavailable | Unknown | Adequate | Unavailable |
| Pulse oximetry probes (reusable) | Unavailable | Unavailable | Unknown | Adequate | Unavailable |
| Functional timer with seconds, n | 1 | Unknown | 0 | 4 | 1 |
| Functional infant scale (digital), n | 1 | 1 | 1 | 0 | 1 |
| Functional infant scale (manual), n | 0 | 0 | 0 | 4 | 0 |
| Gauze | Inadequate | Unavailable | Unknown | Adequate | Adequate |
| Intraosseous (or 22G) needles | Unavailable | Unavailable | Unavailable | Unavailable | Unavailable |
| Kidney bowls | Inadequate | Inadequate | Inadequate | Adequate | Unavailable |
| Lumbar puncture (or 23G) needles | Unavailable | Inadequate | Unavailable | Unavailable | Unavailable |
| Magill’s forceps | Unavailable | Unavailable | Unavailable | Adequate | Unavailable |
| Measuring tape | Inadequate | Inadequate | Inadequate | Adequate | Adequate |
| Neonatal measuring mats | Unavailable | Inadequate | Inadequate | Unavailable | Unavailable |
| Neonatal stethoscopes | Unavailable | Unavailable | Inadequate | Adequate | Unavailable |
| Neonatal thermometers (analogue) | Unavailable | Unavailable | Unavailable | Unavailable | Unavailable |
| Neonatal thermometers (digital) | Unavailable | Inadequate | Inadequate | Unavailable | Unavailable |
| Newborn mittens | Unavailable | Unavailable | Unknown | Unavailable | Unavailable |
| Newborn socks | Unavailable | Unavailable | Unknown | Unavailable | Unavailable |
| Padded boards/splints for neonates | Unavailable | Unavailable | Unknown | Unavailable | Unavailable |
| Sample collection tubes (pus, CSF) | Unavailable | Unavailable | Unavailable | Unavailable | Unavailable |
| Scissors | Unavailable | Unavailable | Inadequate | Adequate | Unavailable |
| Spacer | Unavailable | Unavailable | Unavailable | Unavailable | Unavailable |
| Sterile lancet for heel pricks | Unavailable | Unavailable | Unavailable | Unavailable | Unavailable |
| Sterile surgical blades | Inadequate | Unavailable | Inadequate | Adequate | Unavailable |
| Suction catheters, size 5 | Unavailable | Unavailable | Inadequate | Unavailable | Unavailable |
| Suction catheters, size 6 | Unavailable | Unavailable | Inadequate | Adequate | Unavailable |
| Suction catheters, size 8 or 10 | Unavailable | Unavailable | Unavailable | Unavailable | Unavailable |
| Suture set | Unavailable | Unavailable | Unavailable | Adequate | Unavailable |
| Swabs and/or cotton balls | Adequate | Inadequate | Adequate | Adequate | Inadequate |
| Tourniquet | Unavailable | Unavailable | Unavailable | Unavailable | Unavailable |
| Ultrasound (portable) with probes | Unavailable | Unavailable | Unavailable | Adequate | Unavailable |
| Umbilical cord clamp/sterile ties | Unavailable | Unavailable | Unavailable | Adequate | Unavailable |
| Umbilical vein catheter, size 3.5 or 5 | Unavailable | Unavailable | Unavailable | Unavailable | Unavailable |
| Urinary catheter, size 3, 4, or 5 | Unavailable | Unavailable | Unavailable | Adequate | Unavailable |
| Urine bags (paediatric) | Unavailable | Unavailable | Unavailable | Adequate | Unavailable |
| Urine dipsticks | Adequate | Unavailable | Unavailable | Adequate | Adequate |
| X-ray viewer (any unit) | Unavailable | Unavailable | Unavailable | Unavailable | Unavailable |
| **Resuscitation** |  |  |  |  |  |
| Ventilation bag (neonatal size) | Inadequate | Inadequate | Inadequate | Adequate | Inadequate |
| Face mask (size 0) | Unavailable | Unavailable | Inadequate | Adequate | Unavailable |
| Face mask (size 1) | Unavailable | Inadequate | Inadequate | Adequate | Inadequate |
| Penguin sucker | Inadequate | Inadequate | Inadequate | Adequate | Inadequate |
| Resuscitation mannikin | Inadequate | Unavailable | Inadequate | Adequate | Unavailable |
| T piece resuscitators | Unavailable | Unavailable | Inadequate | Unavailable | Unavailable |
| **Thermal protection** |  |  |  |  |  |
| Functional incubator, n | 1 | 0 | 10 | 29 | 1 |
| Functional radiant heater, n | 2 | 1 | 1 | 20 | 2 |
| Functional temperature monitor^†^, n | 3 | 0 | 0 | 0 | 0 |
| Temperature probe (consumable) | Unavailable | Unavailable | Unavailable | Unavailable | Unavailable |
| Temperature probe (reusable) | Inadequate | Inadequate | Unavailable | Adequate | Unavailable |
| Radiant heater probes | Unavailable | Unavailable | Unavailable | Unavailable | Unavailable |
| Newborn hats/caps | Unavailable | Unavailable | Inadequate | Unavailable | Unavailable |
| Plastic bags/cling film | Unavailable | Unavailable | Unavailable | Unavailable | Unavailable |
| **IV fluid and drug administration** |  |  |  |  |  |
| Adhesive tape | Inadequate | Inadequate | Inadequate | Inadequate | Inadequate |
| IV cannula (22G, 24G, or 26G) | Inadequate | Inadequate | Adequate | Adequate | Inadequate |
| IV infusion set with burette^^^ | Inadequate | Inadequate | Inadequate | Adequate | Inadequate |
| IV infusion stands | Inadequate | Unavailable | Unavailable | Adequate | Unavailable |
| Sterile 19-26G needles/butterfly set | Unavailable | Unavailable | Unavailable | Unavailable | Unavailable |
| Sterile syringes (size 1) | Unavailable | Unavailable | Inadequate | Unavailable | Unavailable |
| Sterile syringes (size 2) | Inadequate | Inadequate | Inadequate | Adequate | Inadequate |
| Sterile syringes (size 5) | Inadequate | Inadequate | Inadequate | Adequate | Inadequate |
| Sterile syringes (size 10) | Inadequate | Inadequate | Inadequate | Adequate | Inadequate |
| Stopcocks (2- or 3-way) | Unavailable | Unavailable | Unavailable | Unavailable | Unavailable |
| Syringe pumps (single phase) | 0 | 0 | 0 | 1 | 3 |
| **Feeding** |  |  |  |  |  |
| Functional breast pump (battery), n | 0 | 0 | 0 | 1 | 0 |
| Breastmilk collection containers | Unavailable | Unavailable | Inadequate | Adequate | Unavailable |
| Breastmilk substitute | Unavailable | Unavailable | Unavailable | Unavailable | Unavailable |
| Feeding bottles | Unavailable | Unavailable | Unavailable | Unavailable | Unavailable |
| Feeding cups | Unavailable | Unavailable | Inadequate | Adequate | Unavailable |
| Feeding syringes | Inadequate | Unavailable | Inadequate | Adequate | Unavailable |
| Nasogastric tubes, size 3.5 with caps | Unavailable | Unavailable | Unavailable | Unavailable | Unavailable |
| Nasogastric tubes, size 4 with caps | Inadequate | Unavailable | Inadequate | Adequate | Unavailable |
| Nasogastric tubes, size 5 with caps | Inadequate | Inadequate | Inadequate | Adequate | Adequate |
| Nasogastric tubes, size 6 with caps | Inadequate | Inadequate | Inadequate | Unavailable | Adequate |
| Nasogastric tubes, size 8 with caps | Unavailable | Unavailable | Unavailable | Unavailable | Unavailable |
| Nasogastric tubes, size 10 with caps | Unavailable | Unavailable | Unavailable | Unavailable | Unavailable |
| **Oxygen therapy** |  |  |  |  |  |
| Functional apnoea monitor, n | 0 | 0 | 0 | 0 | 0 |
| Functional oxygen blender, n | 0 | 0 | 0 | 0 | 0 |
| Functional oxygen concentrator, n | 2 | 1 | 2 | 4 | 2 |
| Functional oxygen cylinder, n | 2 | 3 | 0 | 5 | 2 |
| Functional oxygen flow splitter, n | 0 | 0 | 0 | 1 | 0 |
| Functional oxygen humidifier, n | 2 | 3 | 8 | 4 | 2 |
| Functional respiratory monitor^†^, n | 0 | 0 | 0 | 0 | 0 |
| Functional suction pump (electric), n | 1 | 0 | 0 | 2 | 1 |
| Functional suction pump (manual), n | 0 | 0 | 1 | 0 | 0 |
| Nasal prongs (1 mm) | Adequate | Unavailable | Inadequate | Adequate | Inadequate |
| Nasal prongs (2 mm) | Adequate | Inadequate | Inadequate | Adequate | Inadequate |
| Oxygen cannula | Adequate | Unavailable | Inadequate | Unavailable | Unavailable |
| Oxygen concentrator filter (spare) | Unavailable | Unavailable | Unavailable | Adequate | Unavailable |
| Oxygen tubing | Adequate | Adequate | Unknown | Adequate | Adequate |
| **CPAP and mechanical ventilation** |  |  |  |  |  |
| Functional CPAP flow driver, n | 0 | 0 | 0 | 5 | 0 |
| Functional mechanical ventilator, n | 0 | 0 | 0 | 3 | 0 |
| Functional infant laryngoscope, n | 0 | 0 | 0 | 2 | 0 |
| Functional iSTAT device^‡^, n | 0 | 0 | 0 | 0 | 0 |
| Capnometers | Inadequate | Unavailable | Unavailable | Unavailable | Unavailable |
| CPAP tubing | Unavailable | Unavailable | Unavailable | Adequate | Unavailable |
| CPAP hats to hold in place | Unavailable | Unavailable | Unavailable | Adequate | Unavailable |
| Chest drain set | Inadequate | Unavailable | Unavailable | Unavailable | Unavailable |
| Chest drain tubing, water seal bottle | Inadequate | Unavailable | Unavailable | Unavailable | Unavailable |
| Distilled water | Unavailable | Unavailable | Unavailable | Adequate | Unavailable |
| Endotracheal tubes, size 2 | Unavailable | Unavailable | Unknown | Unavailable | Unavailable |
| Endotracheal tubes, size 2.5 | Unavailable | Unavailable | Unknown | Inadequate | Unavailable |
| Endotracheal tubes, size 3 | Unavailable | Unavailable | Unknown | Unavailable | Unavailable |
| Endotracheal tubes, size 3.5 | Unavailable | Unavailable | Unknown | Unavailable | Unavailable |
| iSTAT cartridges^‡^ | Inadequate | Unavailable | Unavailable | Unavailable | Unavailable |
| Laryngoscope light bulbs (spare) | Unavailable | Unavailable | Unavailable | Inadequate | Unavailable |
| Transilluminators | Unavailable | Unavailable | Unavailable | Unavailable | Unavailable |
| **Phototherapy** |  |  |  |  |  |
| Functional phototherapy unit^¶^, n | 2 | 2 | 2 | 6 | 2 |
| Functional bilirubinometer^§^, n | 0 | 0 | 0 | 0 | 0 |
| Functional photo-light meter, n | 1 | 0 | 0 | 0 | 1 |
| Bilirubinometer test strips | Unavailable | Unavailable | Unavailable | Unavailable | Unavailable |
| Eye patches/eye shields | Unavailable | Unavailable | Unavailable | Adequate | Unavailable |
| Icterometer | Unavailable | Unavailable | Unavailable | Unavailable | Unavailable |
| Phototherapy LED bulb- spare | Unavailable | Unavailable | Inadequate | Unavailable | Unavailable |
| Phototherapy fluorescent bulb- spare | Unavailable | Unavailable | Inadequate | Unavailable | Unavailable |
| Exchange transfusion sets (any unit) | Unavailable | Unavailable | Unavailable | Unavailable | Inadequate |

CPAP=continuous positive airway pressure. CSF=cerebrospinal fluid. ECG=electrocardiogram. G=gauge. IV=intra-venous. LED=light-emitting diode. mL=millilitre. mm=millimetre. SpO_2_=peripheral capillary oxygen saturation. ^*^Patient monitor including at least 3 of: blood pressure, ECG, heart rate, respiratory rate, SpO_2_, temperature. ^^^IV infusion set with 100-150 mL burette. ^†^Device that continuously monitors temperature or respiratory rate. ^‡^iSTAT device and cartridges for blood gas analysis. ^¶^Phototherapy unit with LED or fluorescent bulbs. ^§^Traditional or transcutaneous bilirubinometer.

# **References**

1. Wilkinson T, Sculpher M, Claxton K, Revill P, Briggs A, Cairns J, et al. The International Decision Support Initiative Reference Case for Economic Evaluation: An Aid to Thought. Value Health. 2016; 19:921–8.

2. Medvedev MM, Tumukunde V, Mambule I, Tann CJ, Waiswa P, Canter RR, et al. Operationalising kangaroo Mother care before stabilisation amongst low birth Weight Neonates in Africa (OMWaNA): protocol for a randomised controlled trial to examine mortality impact in Uganda. Trials. 2020;21:126.
