# Supplementary material for: Process and costs for readiness to safely implement immediate kangaroo mother care: a mixed methods evaluation from the OMWaNA trial at five hospitals in Uganda
Source: BMC Health Serv Res. 2023 Jun 10;23:613. doi: 10.1186/s12913-023-09624-z (PMC10257176; doi:10.1186/s12913-023-09624-z)

Neonatal unit floor plans pre- and post-renovation at the five hospitals in Uganda

Entebbe Regional Referral Hospital: floor plan pre-renovation

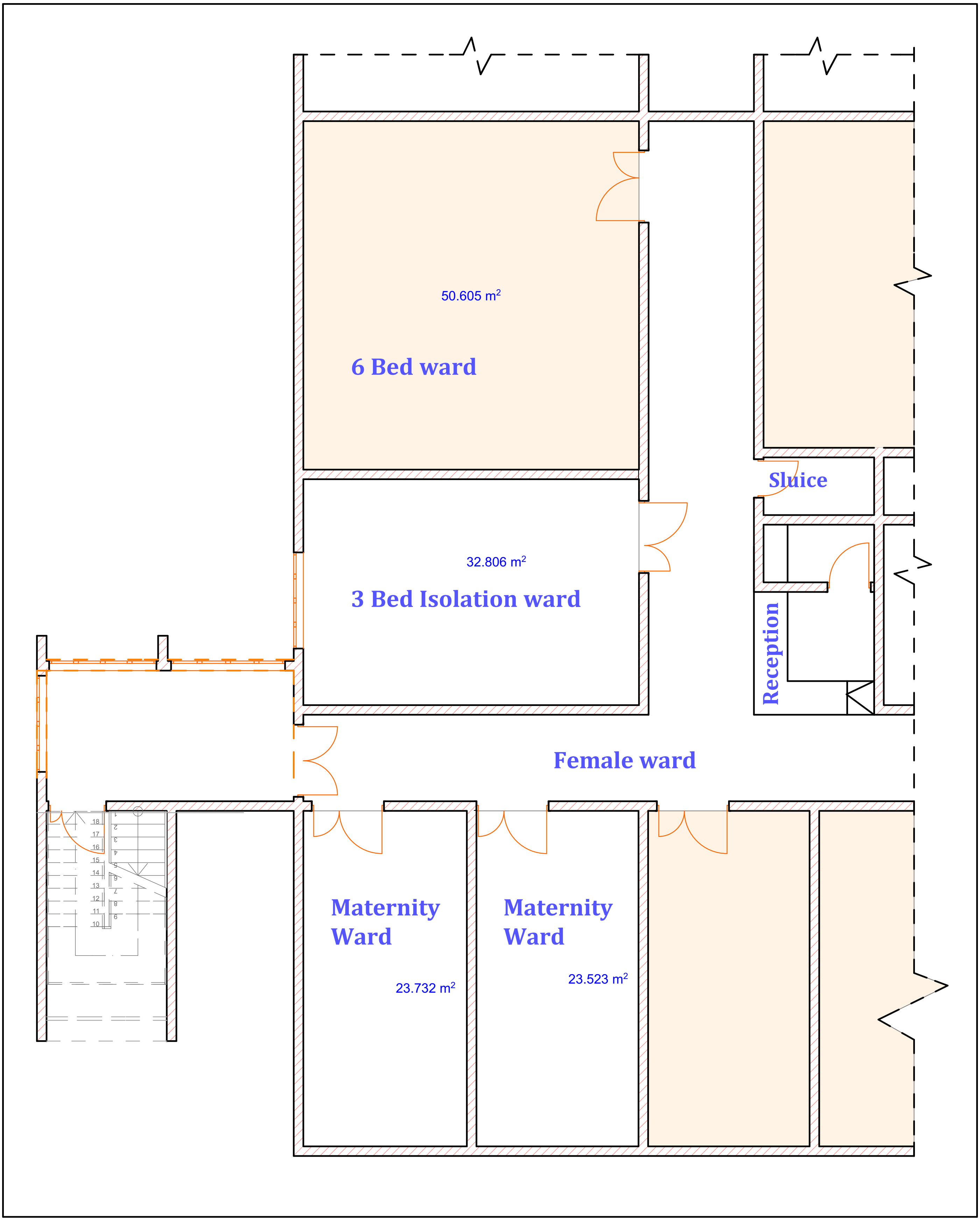

Entebbe Regional Referral Hospital: floor plan post-renovation

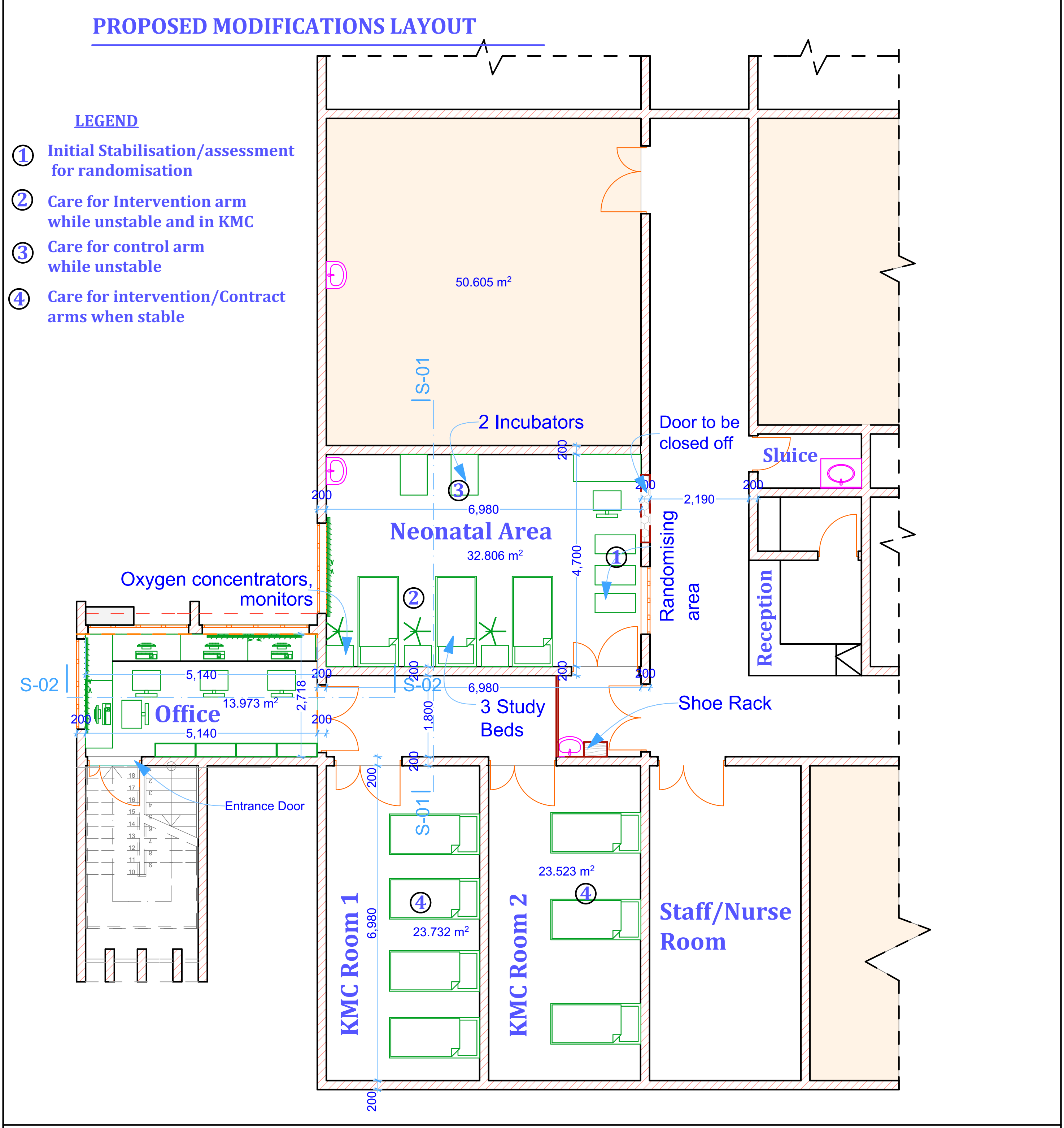

drawing title				project
PROPOSED PLAN				PROPOSED NEONATAL MODIFICATION WORKS
issue type: CONSTRUCTION		scale: 1:100	paper size: A	project address: ENTEBBE HOSPITAL (GRADE A)
project no.	discipline	drawing no.	revision	client: MRC/UVRI & LSHTM UNIT ON AIDS

MRC/UVRI and LSHTM Uganda Research Unit

MRC Medical Research Council

Uganda Virus Research Institute

LONDON SCHOOL of HYGIENE &TROPICAL MEDICINE

Iganga District Hospital: floor plan pre-renovation

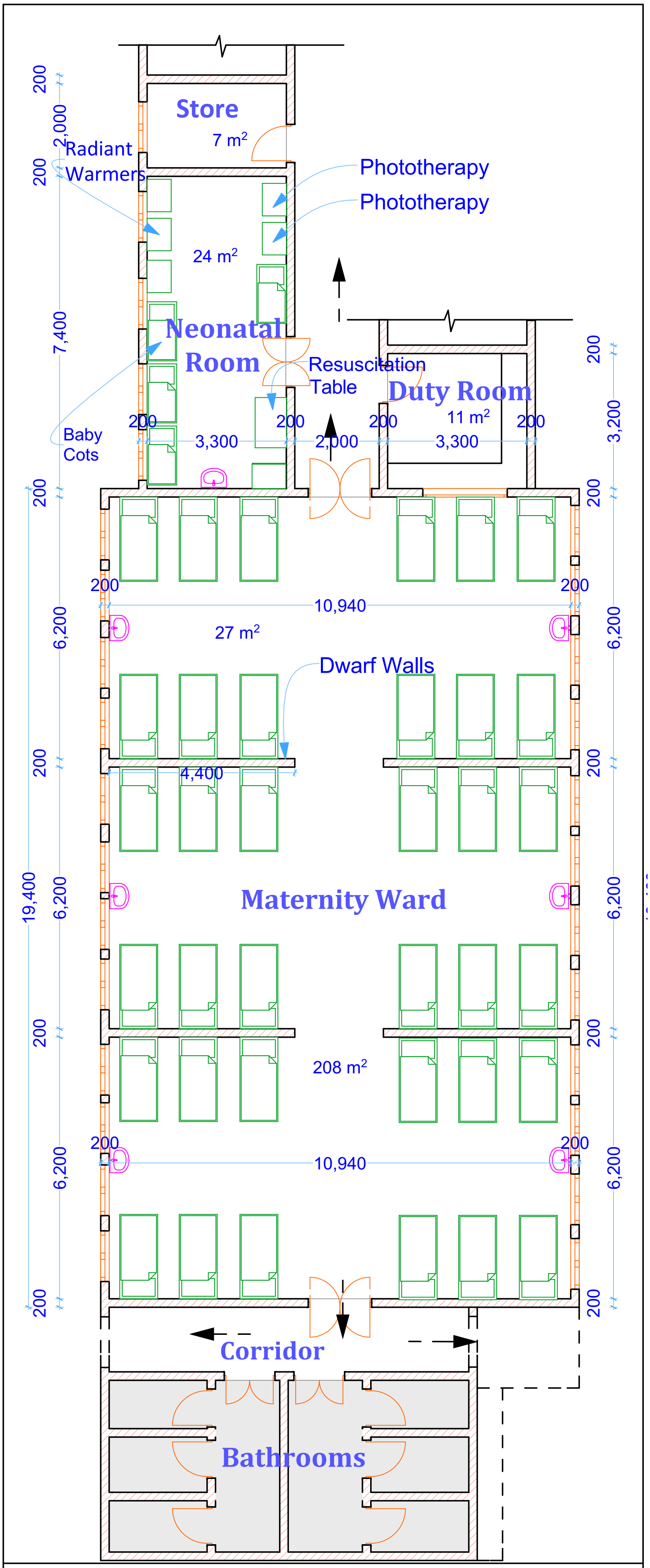

drawing title<br>PROPOSED PLAN				project<br>PROPOSED NEONATAL MODIFICATION WORKS
issue type<br>**CONSTRUCTION**		scale<br>**1:100**	paper size<br>**A**	project address<br>IGANGA HOSPITAL
project no.	discipline	drawing no.	revision	client<br>MRC/UVRI & LSHTM UNIT ON AIDS

Iganga District Hospital: floor plan post-renovation

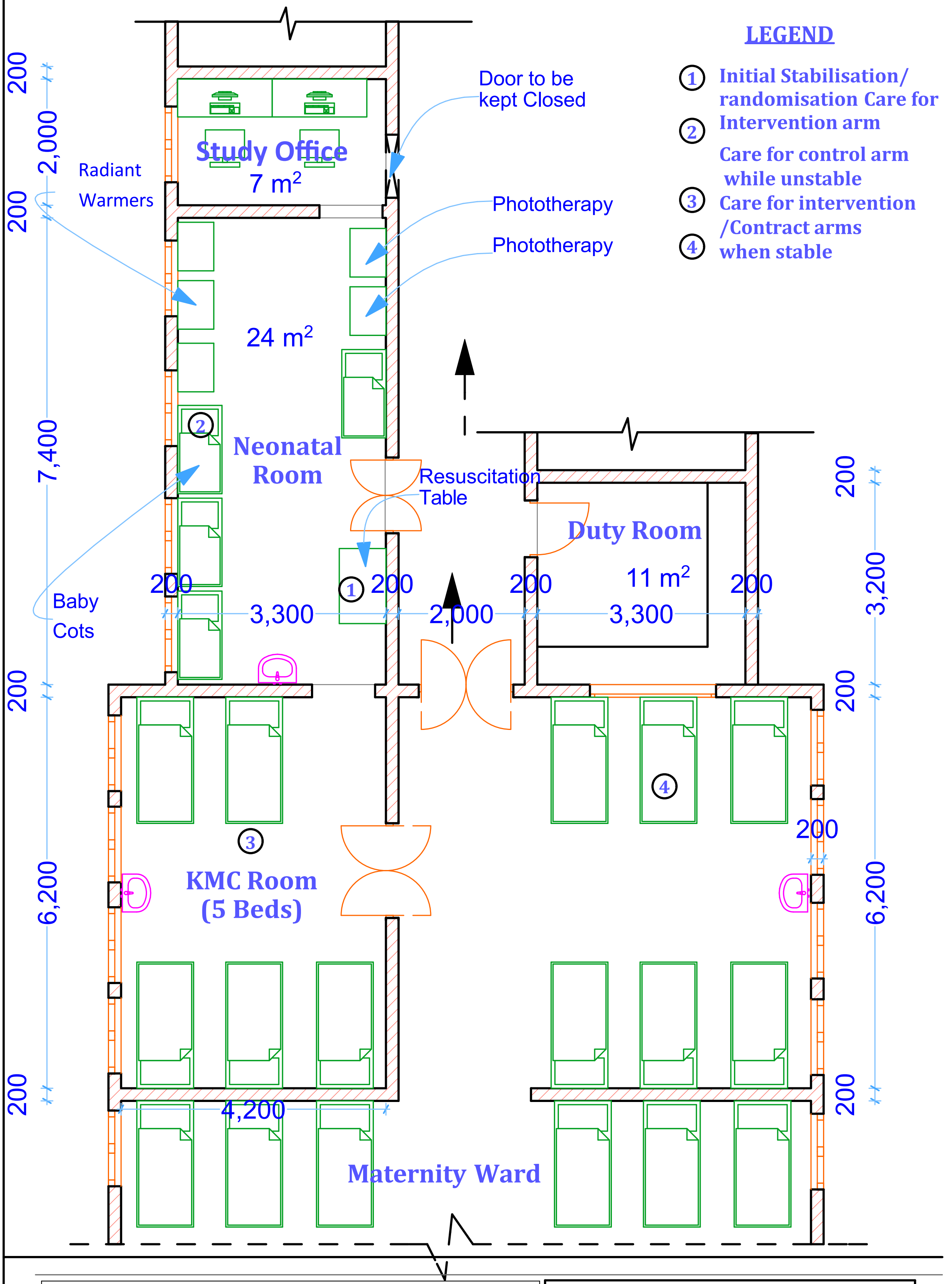

drawing title			
PROPOSED PLAN			
issue type: **CONSTRUCTION**		scale: **1:100**	paper size: **A**
project no.	discipline	drawing no.	revision

project: **PROPOSED NEONATAL MODIFICATION WORKS**
project address: **IGANGA HOSPITAL**
client: **MRC/UVRI & LSHTM UNIT ON AIDS**

Jinja Regional Referral Hospital: floor pre-renovation

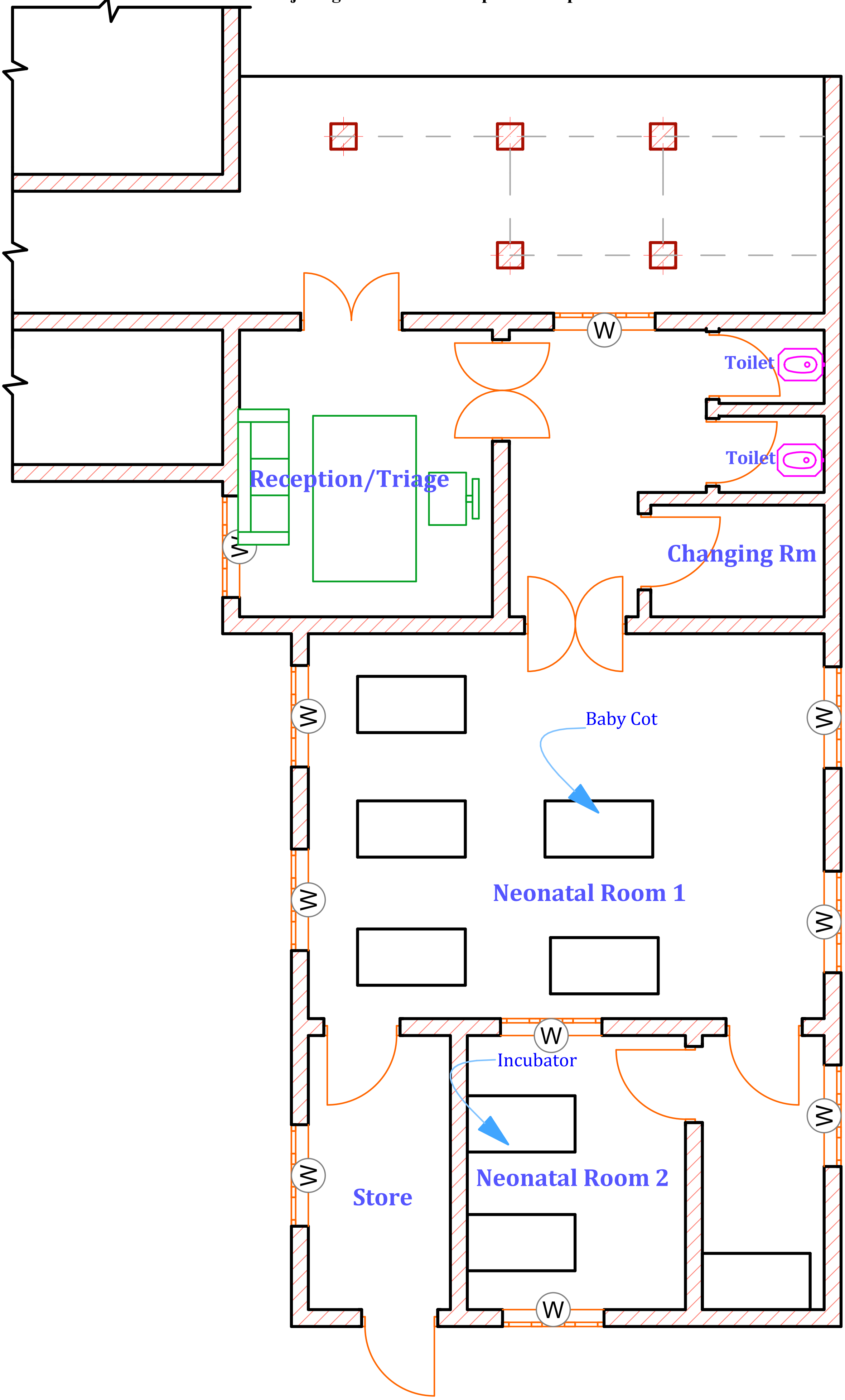

Jinja Regional Referral Hospital: floor plan post-renovation

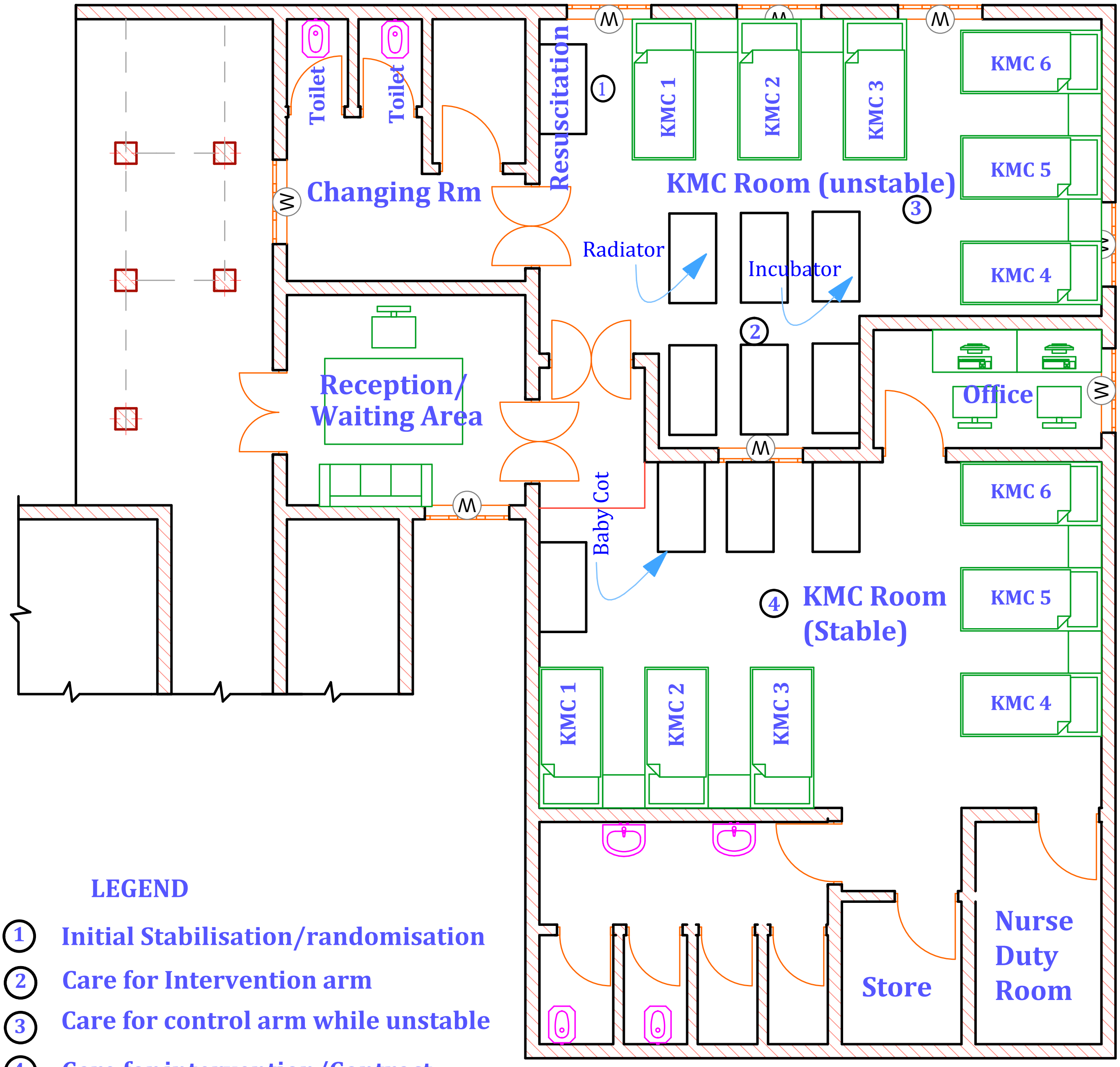

LEGEND

1. Initial Stabilisation/randomisation
2. Care for Intervention arm
3. Care for control arm while unstable
4. Care for intervention/Contract arms when stable

Kawempe National Referral Hospital: floor plan pre-renovation

drawing title
Existing Layout

issue type
Rehabilitation Works

scale
1:100

paper size
A

project no.

discipline

drawing no.

revision

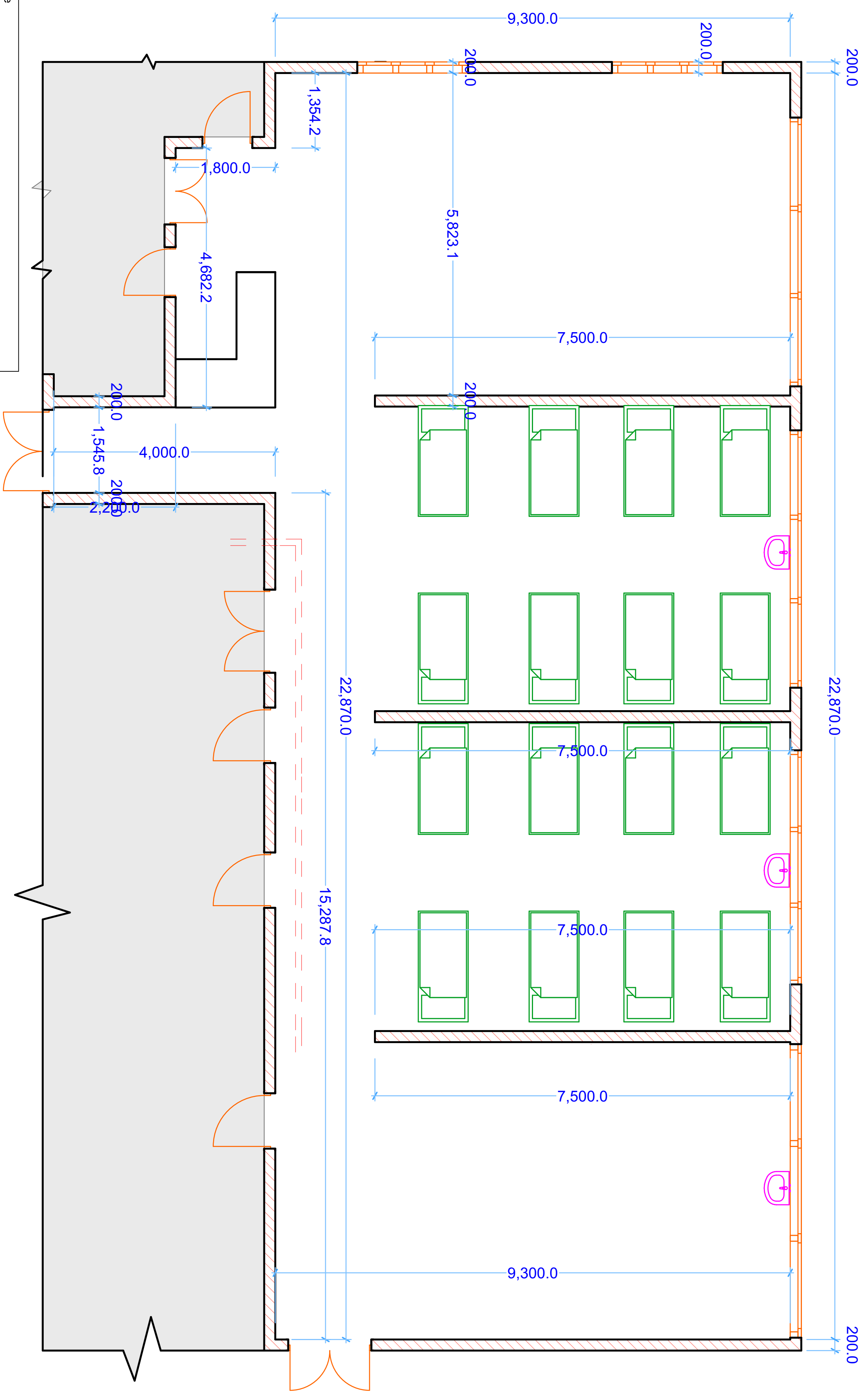

Kawempe National Referral Hospital: floor plan post-renovation

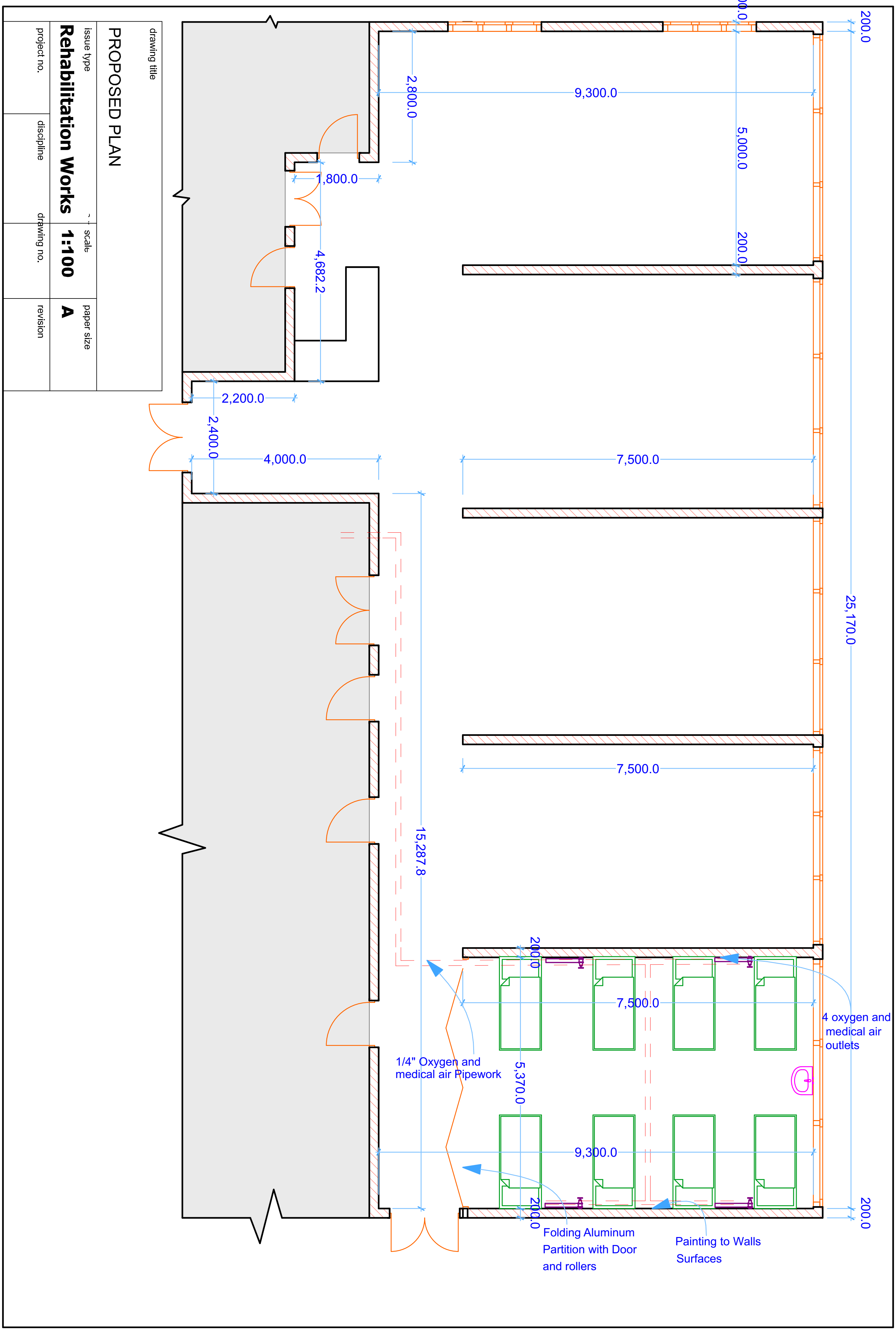

Masaka Regional Referral Hospital: floor plan pre-renovation

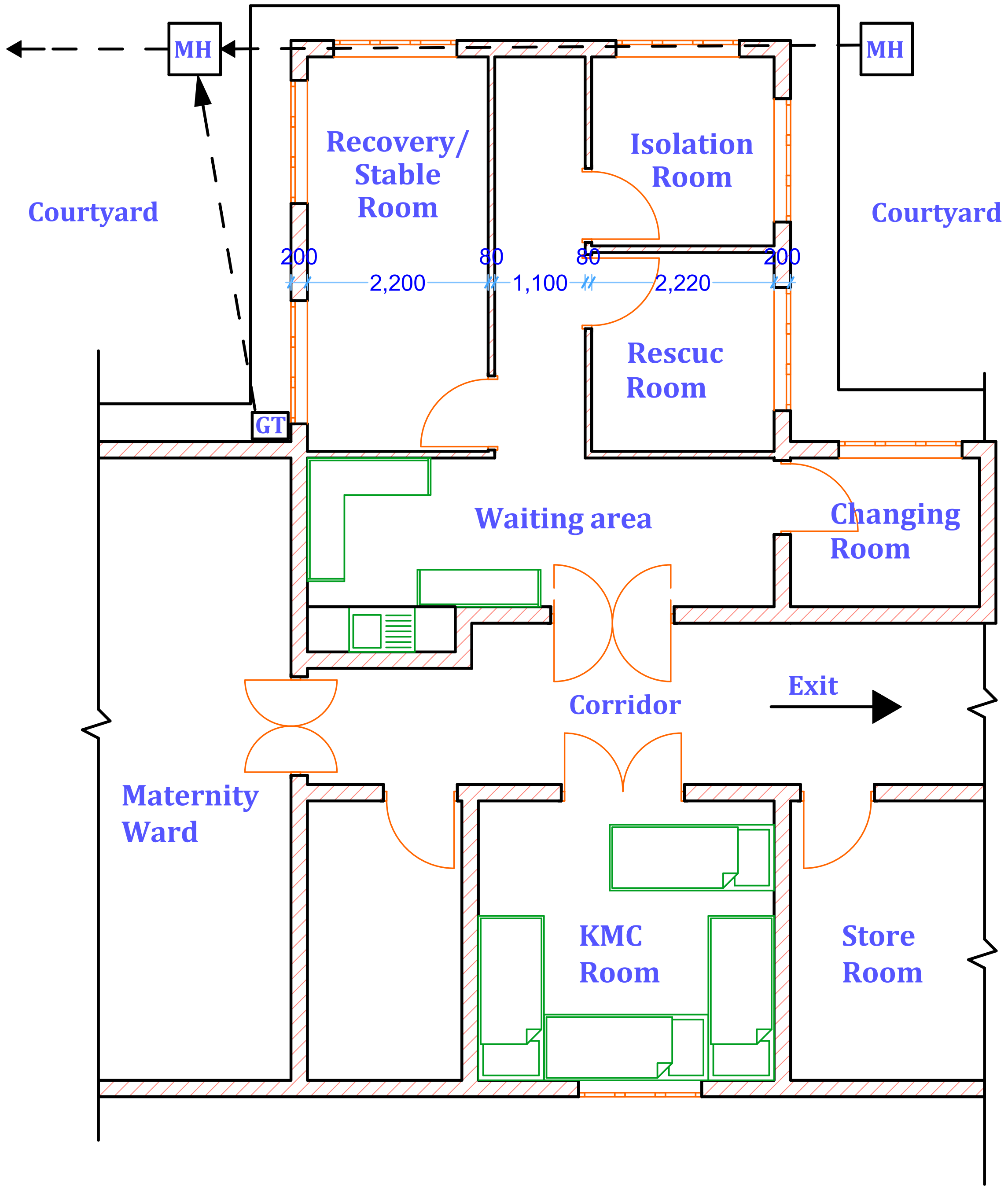

drawing title				project
EXISTING FLOOR LAYOUT				PROPOSED NEONATAL MODIFICATION WORKS
issue type<br>**CONSTRUCTION**		scale<br>**1:100**	paper size<br>**A**	project address<br>MASAKA HOSPITAL
project no.	discipline	drawing no.	revision	client<br>MRC/UVRI & LSHTM UNIT ON AIDS

Masaka Regional Referral Hospital: floor plan post-renovation

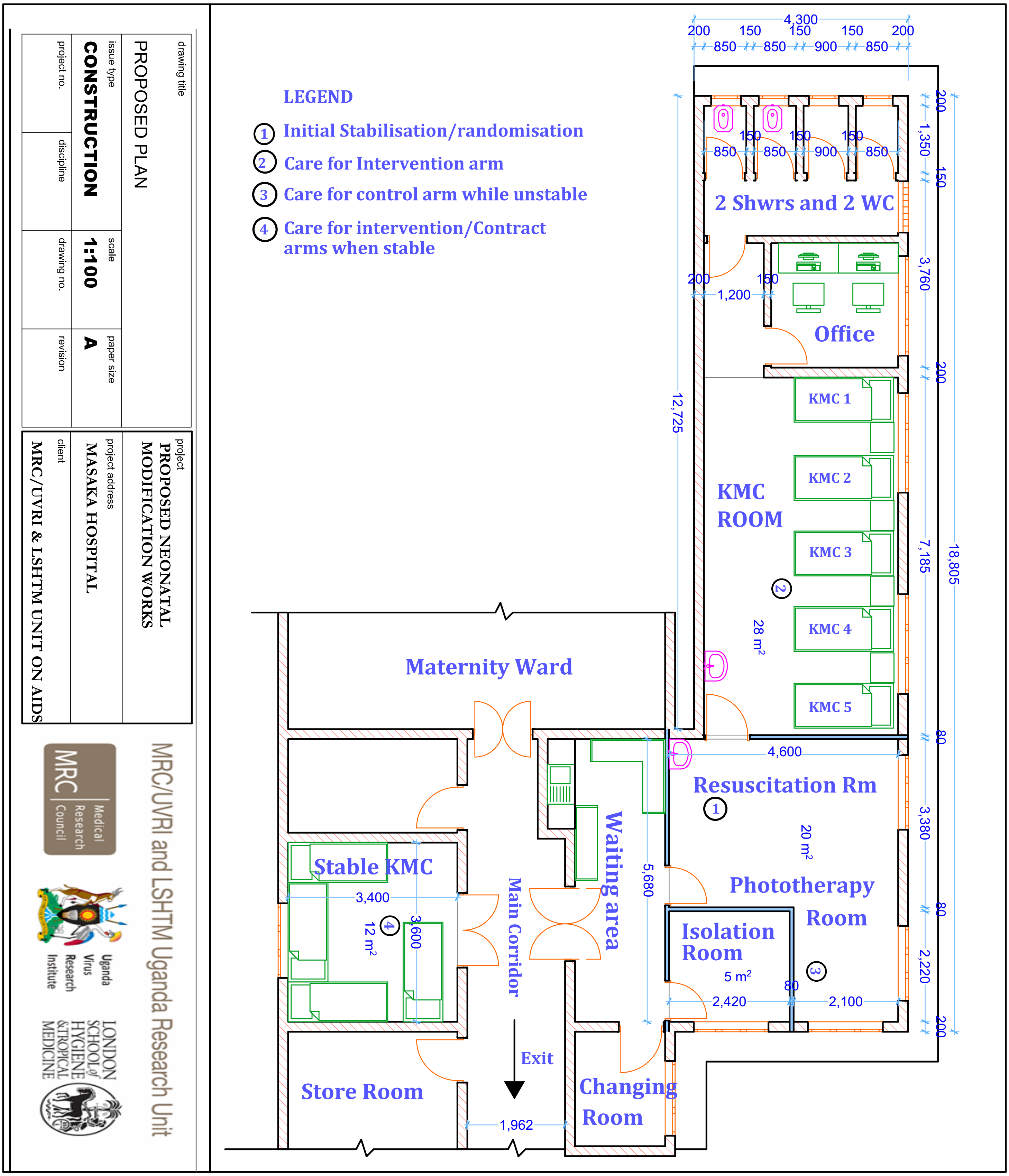

Supplement: Supplementary file 3 — Additional file 3. Neonatal unit floor plans pre- and post-renovation at the five hospitals in Uganda. [file 12913_2023_9624_MOESM3_ESM.pdf]
