# Supplementary material for: Process and costs for readiness to safely implement immediate kangaroo mother care: a mixed methods evaluation from the OMWaNA trial at five hospitals in Uganda
Source: BMC Health Serv Res. 2023 Jun 10;23:613. doi: 10.1186/s12913-023-09624-z (PMC10257176; doi:10.1186/s12913-023-09624-z)
Supplement: Supplementary file 4 — Additional file 4. Author reflexivity statement for equity in global health research. [file 12913_2023_9624_MOESM4_ESM.docx]

# **Author reflexivity statement for equity in global health research**

1. **How does this study address local research and policy priorities?**

The OMWaNA study was designed in close partnership between researchers from the Medical Research Council/Uganda Virus Research Institute (MRC/UVRI) & LSHTM Uganda Research Unit, Makerere University (Uganda), and the London School of Hygiene & Tropical Medicine (UK). A World Health Organisation-led trial reported reduced mortality at 28 days among newborns weighing 1000-1799g who received immediate KMC (iKMC) relative to those who received standard care, prompting calls for widespread adoption of this intervention; evidence is needed regarding the process and costs of implementing iKMC and how these vary across contexts. The results of this study provide evidence to inform investment requirements and decisions about if, where, and how to implement iKMC, particularly in low- and middle-income countries where neonatal intensive care is often unavailable. Further, this study aligns with the overarching priorities of the Ugandan Ministry of Health, which is working to achieve national coverage of KMC in all health facilities.

1. **How were local researchers involved in study design?**

VT, CKN, IM, KK, PW, and EEK are local researchers with extensive in-country experiences. JEL was born in Uganda and has more than 25 years of experience in the design and evaluation of integrated maternal and newborn services at scale, especially in sub-Saharan Africa. The study also involves high-income country researchers with extensive experience in conducting and supervising research studies in Uganda and other low- and middle-income countries (MMM, GG, CJT, DE, EA, CP).

1. **How has funding been used to support the local research team?**

This project has been used to support four early career, Ugandan researchers (VT, CKN, IM, KK) in full-time roles within the OMWaNA project. VT is being supported by the senior authorship team to pursue a PhD focused on iKMC implementation in Uganda, embedded within the OMWaNA trial, through the LSHTM/MRC collaborative programme. CKN is being supported by the senior authorship team to develop research skills and build the foundational groundwork for a PhD fellowship application. IM is nearing completion of a part-time PhD program at the Malawi-Liverpool Wellcome Trust Research Programme, which he has been supported to continue whilst working on the OMWaNA trial in Uganda. KK is currently pursuing a part-time PhD through the LSHTM/MRC collaborative programme, which he has been supported to continue whilst working as a health economist at the MRC/UVRI & LSHTM Uganda Research Unit.

1. **How are research staff who conducted data collection acknowledged?**

All researchers who conducted data collection were included as authors.

1. **Do all members of the research partnership have access to study data?**

All members of the research partnership have access to study data.

1. **How was data used to develop analytical skills within the partnership?**

Local partners were involved throughout the process of data collection and data analysis. EEK, CP, and GG provided guidance to MMM and CKN on the analysis of costing data. This research partnership is ongoing, with several capacity building workshops for quantitative and qualitative analyses and scientific writing scheduled in the coming months.

1. **How have research partners collaborated in interpreting study data?**

Monthly virtual meetings were held with the research team to discuss data collection, data analysis, the preliminary findings, and data interpretation.

1. **How were research partners supported to develop writing skills?**

Post-doctoral early career researcher MMM led the writing of this manuscript, with regular input from the in-country research team and the senior authors. Local pre-doctoral early career researchers (VT, CKN, IM, KK) on the authorship team were supported by senior academics to develop and refine their writing skills. CKN attended a LSHTM webinar on scientific writing and the publication process for early career researchers.

1. **How will research products be shared to address local needs?**

This article will be published as open access. We have submitted the study abstract for presentation at an upcoming international meeting, which will be attended by a wide variety of stakeholders based in both high-income and low- and middle-income countries. The study results will also be disseminated in Uganda through a series of meetings and workshops with stakeholders from the Ministry of Health’s Newborn Steering Committee, the Uganda Paediatric Association, the MRC/UVRI & LSHTM Uganda Research Unit, Makerere University, and the five study hospitals.

1. **How is the leadership, contribution, and ownership of this work by LMIC researchers recognized within the authorship?**

Six Ugandan researchers are included in the authorship team (VT, CKN, IM, KK, PW, EEK). EEK worked as part of the senior authorship team in developing this manuscript and her contribution has been recognised as joint senior author. VT and CKN have been recognised at joint second authors given their contributions to data collection, analysis, and interpretation of the results. IM, KK, and PW have been recognised as fifth, sixth, and seventh authors, respectively. We acknowledge, however, that seven authors are based in high-income countries, including the first author (MMM). This paper was led by MMM as part of her postdoctoral fellowship work, which is supervised by JEL. The decision about who would lead this manuscript (as first, joint second, and joint senior authors) was made collectively by the full research team. All local researchers who contributed to this paper have been included as co-authors.

1. **How have early career researchers across the partnership been included within the authorship team?**

Five early career researchers are included within the authorship team. Four of these authors are Ugandan (VT, CKN, IM, KK), and one is based in a high-income country (MMM). MMM co-conceived the study, analysed the HFA data, contributed to the analysis of costing data, interpreted the study data, led the writing and revision of this manuscript, and acquired funding to support this work. VT collected the HFA data and contributed to data interpretation and overall study coordination. CKN collected the costing data and contributed to cost data analysis and data interpretation. IM and KK supported data collection and contributed to data interpretation.

1. **How has gender balance been addressed within the authorship?**

The first author and all three joint senior authors are female. Nine authors are female (MMM, CKN, GG, CJT, DE, EA, EEK, CP, JEL) and four authors are male (VT, IM, KK, PW).

1. **How has the project contributed to training of LMIC researchers?**

Research funding leveraged as part of this project is currently supporting the employment of three early career Ugandan researchers (VT, CKN, IM), all of whom are included in the authorship team. Funds from this project are supporting VT to pursue a PhD in the LSHTM/MRC collaborative programme, including travel to and accommodation in London in January-February 2023 to complete the Statistical Methods in Epidemiology course and practical sessions on data analysis at LSHTM under the guidance of CJT, JEL, and other senior academics. CKN is being supported by project funds to develop research skills and build the foundational groundwork for a PhD fellowship application in the coming years. CKN attended an advanced Excel training course and a webinar for early career researchers on scientific writing and the publication process at LSHTM.

1. **How has the project contributed to improvements in local infrastructure?**

As described in the manuscript, substantial expansion of neonatal care capacity and infrastructure improvements were required at all five hospitals to safely implement iKMC. The renovation process included the establishment of neonatal units with adult beds for KMC practice, through remodelling of existing units, repurposing of space, or construction of a new unit. Renovations also included offices for clinical staff, bathrooms and toilets for mothers and other caregivers, and sinks in clinical areas to provide an optimal environment for infection prevention and control. All hospitals were also provided with essential equipment and supplies to support the provision of KMC and small and sick newborn care.

1. **What safeguarding procedures were used to protect local study participants and researchers?**

Ethical approval for the study was obtained from the Research Ethics Committees of the Uganda Virus Research Institute (GC/127/19/06/717), LSHTM (16972), and the Uganda National Council of Science and Technology (HS 2645). The health facility assessment and costing data did not require individual informed consent.

The OMWaNA study was monitored by the Clinical Studies Support Office at the MRC/UVRI & LSHTM Uganda Research Unit. The monitoring team comprised of study coordinators and study managers from the various trials that were currently running within the unit. One dedicated study monitor, independent of the research team, oversaw progress and ensured that the study was conducted, and data were handled in accordance with standard operating procedures, the protocol, Good Clinical Practice, and applicable ethical and regulatory requirements.
